# Key Amino Acid Residues of the Agt1 Transporter for Trehalose Transport by *Saccharomyces cerevisiae*

**DOI:** 10.3390/jof10110781

**Published:** 2024-11-11

**Authors:** Anqi Chen, Yuhan Cheng, Liushi Meng, Jian Chen

**Affiliations:** 1 Science Center for Future Foods, Jiangnan University, Wuxi 214122, Chinajchen@jiangnan.edu.cn (J.C.); 2 Key Laboratory of Industrial Biotechnology of Ministry of Education, School of Biotechnology, Jiangnan University, Wuxi 214122, China; 3 Jiaxing Synbiolab Technology Co., Ltd., Jiaxing 314000, China; 4 State Key Laboratory of Food Science and Resources, Jiangnan University, Wuxi 214122, China

**Keywords:** trehalose, Agt1, *Saccharomyces cerevisiae*, molecular dynamics, site-directed mutagenesis, stress resistance

## Abstract

Trehalose is crucial for the stress resistance of *Saccharomyces cerevisiae*, primarily through its stabilization of proteins and membranes. The Agt1 transporter, a member of the Major Facilitator Superfamily, mediates trehalose uptake, a key process for maintaining cellular integrity under stress. Despite its importance, the molecular mechanisms of Agt1-mediated trehalose transport remain underexplored. In this study, we expressed and purified the trehalase enzyme TreA from *E. coli* to develop reliable trehalose assays. We screened 257 wild *S. cerevisiae* isolates, identifying strains with enhanced trehalose transport capacities. Comparative analyses, including structural modeling and molecular docking, revealed that specific Agt1 variants exhibited significantly higher transport efficiency, influenced by key residues in the transporter. Molecular dynamics simulations and steered molecular dynamics provided further insights, particularly into the role of the Agt1 channel head region in substrate recognition and binding. Site-directed mutagenesis validated these findings, showing that mutations at critical residues, such as 156Q, 164L, 256Q, 395E, 396R, and 507Y significantly reduced transport activity, while 137Q, 230T, and 514 N increased efficiency under certain conditions.

## 1. Introduction

**Figure 1:**
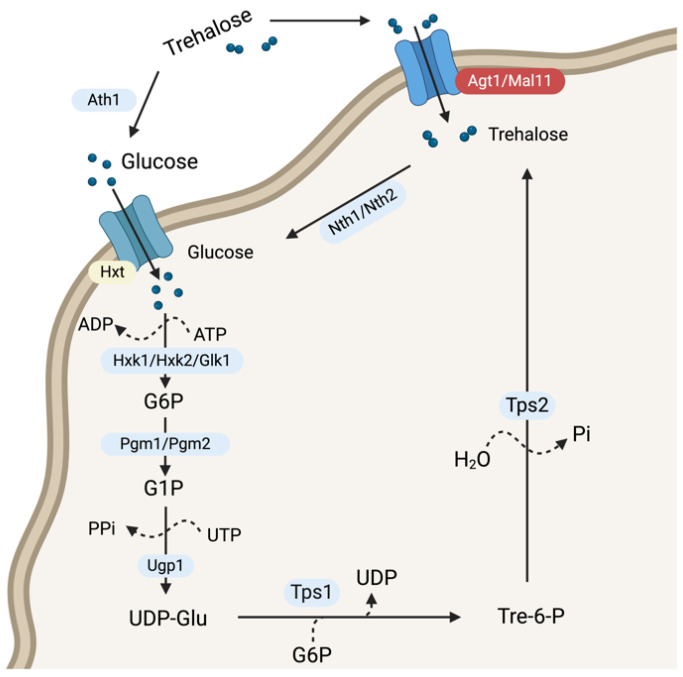
Trehalose metabolism in *S. cerevisiae.* Major metabolites, enzymes/proteins, and other reactants/products are indicated. Tps3 and/or Tsl1 have proposed roles in supporting Tps1 and/or Tps2 function, though the mechanism is not clearly understood. The Agt1 transporter is also known as Mal11. G6P, Glucose-6-phosphate; G1P, Glucose-1-phosphate; UDP, Uridine 5′-diphosphate; UDP-Glu, Uridine 5′-disphophate glucose; Tre-6-P, trehalose-6-phosphate.

Trehalose, a non-reducing disaccharide composed of two glucose molecules linked by an α,α-1,1-glycosidic bond, plays a critical role in the biology of *Saccharomyces cerevisiae*, particularly in its response to environmental stresses [1,2]. As both a reserve carbohydrate and a stabilizer of proteins and cellular membranes, it provides protection during conditions like desiccation and freeze-thaw stress [3,4]. Trehalose is synthesized from glucose via the trehalose-6-phosphate pathway, regulated by Tps1 and Tps2, while its degradation is catalyzed by Nth1, Nth2, and Ath1 (Figure 1) [5]. Accumulation of trehalose is especially important when yeast cells enter the stationary phase or encounter stressful environments, as it helps preserve the structural integrity of proteins and lipids [6]. This makes trehalose metabolism a key focus in industrial biotechnology, particularly in enhancing the stress tolerance of yeast strains used in fermented beverage production.

Transport of trehalose across cellular membranes is largely mediated by Agt1, a member of the Major Facilitator Superfamily (MFS), which co-transports trehalose along with protons to mobilize energy reserves and protect cells from stress [7]. Agt1 is also capable of transporting other α-glucosides like maltose, contributing to its broad substrate specificity [8,9,10]. Despite its well-established role, the molecular mechanisms underlying Agt1-mediated transport remain partially understood, particularly, amino acid sequence variations among yeast strains affect its transport efficiency and specificity [11].

To explore this, we screened a collection of 257 *S. cerevisiae* strains, including two commonly used laboratory strains, isolates from commercial suppliers involved in various fermented food and beverage production, and wild isolates from environments such as vineyards and breweries. This diverse collection provided the necessary genetic variability to identify natural variants with enhanced trehalose transport capabilities, which are critical for improving yeast strains for industrial applications. The regulation of trehalose transport, primarily mediated by Agt1, is of great interest due to its unique role. For instance, strains like S288C, which lacks a functional Agt1, show growth disadvantages on several carbon sources [12,13,14,15,16,17]. Agt1 operates via a co-transport mechanism, moving α-glucoside along with protons, which has been shown to enhance trehalose-related stress resistance when *AGT1* is constitutively expressed [18,19,20]. Advances in AI technology and molecular dynamics tools, such as AlphaFold2 and Schrödinger, provide new opportunities to elucidate the transport mechanism of Agt1 at the molecular level [21,22,23,24,25,26,27].

In this study, we aimed to investigate key amino acids in Agt1 that are crucial for trehalose transport in *S. cerevisiae*. After expressing the trehalase enzyme (TreA) from *E. coli*, we screened the wild isolates for high-trehalose transport activity. Through gene sequencing and structural modeling, we identified key residues involved in substrate binding and transport and further mutated these residues to assess their functional importance. The efforts culminated in the development of yeast strains with elevated trehalose transport abilities, offering new insights into trehalose metabolism and its industrial applications.

## 2. Materials and Methods

### 2.1. Yeast Strains and Growth Conditions

A total of 257 wild *S. cerevisiae* strains, a kind gift from Cornell University, were used in this study, comprising two widely used laboratory strains, isolates from commercial suppliers involved in fermented food and beverage production and wild strains collected from diverse environments such as vineyards, breweries, and natural habitats across the globe. Yeast cells were grown in minimal medium (YNB: 0.67% *w*/*v* yeast nitrogen base with ammonium sulfate and 2% *w*/*v* carbon sources) or rich medium (YP: 2% *w*/*v* bactopeptone, 1% *w*/*v* yeast extract, and 2% *w*/*v* carbon sources). For transformant selection, a synthetic complete medium lacking uracil (SC-URA) was used. Media were solidified with 2% *w*/*v* agar when needed. Cell density was measured at 600 nm using a Gensys 6 UV-Vis spectrophotometer (Thermo Fisher Scientific, Waltham, MA, USA).

### 2.2. Yeast Strain Construction

The strains and primers used in this study are detailed in Supplementary Tables S1 and S3. Gene deletions were performed by transforming yeast with PCR products from deletion cassettes, which were constructed using plasmids pFA6a-kanMX, pAG32, and pAC372 (Supplementary Table S2). The deletion cassettes included 40 bp flanking sequences homologous to regions surrounding the target genes, allowing integration into the yeast genome via homologous recombination. Transformants were selected on antibiotic-containing media, and correct gene deletions were confirmed via PCR. For Agt1 mutants, site-directed mutagenesis was used to introduce alanine substitutions into key amino acid residues. The p416-S288C-Agt1 plasmid, harboring the *AGT1* gene, was used as the template for mutagenesis. Mutant plasmids were sequenced to verify changes before transformation into the *ura3*Δ0 auxotrophic strain.

### 2.3. Plasmid Construction

Plasmids were constructed using pRS-series shuttle vectors driven by the *TDH3* promoter and containing the *CYC1* 3′ UTR [28,29]. PCR were generated with SpeI and XhoI restriction sites for cloning into vector backbones. The ligation reactions were transformed into *E. coli* DH5α, and positive clones were selected on LB agar containing the appropriate antibiotics. Successful plasmid constructions were verified by Sanger sequencing and used for subsequent yeast transformations.

### 2.4. Trehalase Expression and Purification

The *E. coli* trehalase gene (*treA*) was cloned into the pET-28a (+) expression vector using primers with NdeI and XhoI restriction sites. Following confirmation of the clone by sequencing, the plasmid was transformed into *E. coli* BL21(DE3) cells for protein expression. Protein expression was induced with 1 mM IPTG at 16 °C for 16 h in 2xYT medium. Cells were harvested by centrifugation using an Avanti J-26 XPI high-speed centrifuge (Beckman Coulter, Brea, CA, USA), resuspended in 50 mM phosphate buffer (pH 5), and lysed using an EmulsiFlex-C5 high-pressure homogenizer (Avestin, Ottawa, ON, Canada). The trehalase protein was purified using a Ni-NTA agarose column (Qiagen, Hilden, Germany), and its purity was assessed using SDS-PAGE. Protein concentration was determined using the Pierce™ BCA Protein Assay Kit (Thermo Fisher Scientific, USA).

### 2.5. Trehalose Transport Assay

To quantify trehalose uptake, yeast cells were grown in the YPD to mid-log phase, and two aliquots, each containing 10 OD units, were harvested. Intracellular trehalose levels were immediately measured in one aliquot to establish a baseline, while the other aliquot was treated with 1 g/L trehalose for 2 h to allow for trehalose transport. After treatment, intracellular trehalose levels were measured again. Trehalose quantification was performed as described by [30]. Cell density was measured at 600 nm using a Genesys 6 UV-Vis spectrophotometer (Thermo Fisher). The harvested cells were washed with ice-cold water and resuspended in 250 μL of 0.25 M sodium carbonate. For the assay, cells were boiled at 95 °C for 4 h with occasional agitation to degrade any residual glucose while preserving trehalose. After cooling, 150 μL of 1 M acetic acid and 600 μL of 0.2 M sodium acetate were added. A 350 μL aliquot was transferred to a fresh tube, and 5 μL of 70 U/mL trehalase (expressed from *E. coli*) was added. The mixture was incubated overnight at 37 °C, then centrifuged at maximum speed for 3 min. Finally, 200 μL of the supernatant was used to measure glucose liberated from trehalose using a commercial glucose assay (Jiancheng Bioengineering, Nanjing, China).

### 2.6. Agt1 Sequence Analysis

The *AGT1* gene from various yeast strains was PCR amplified using primers based on the *S. cerevisiae* S288C reference sequence (*Saccharomyces* Genome Database, SGD) [31]. Genomic DNA was extracted using the DNeasy Blood & Tissue Kit (Qiagen, Germany), and PCR products were sequenced. The sequences were aligned and analyzed using the MUSCLE algorithm in MEGA 11 software [32,33]. To improve alignment accuracy, the N-terminal 19 amino acids and C-terminal 16 amino acids were excluded from the analysis.

### 2.7. Agt1 Structure Modeling and Docking

Amino acid sequences of Agt1 from selected strains were submitted to the ColabFold 15.5 tool (AlphaFold, DeepMind, London, UK) for three-dimensional structure predictions. The highest-confidence models, based on PLDDT scores, were refined using the Schrödinger Suite (Schrödinger, New York, NY, USA). Trehalose (Compound CID: 7427) was converted into a three-dimensional conformation using LigPrep (Schrödinger, USA). Sitemap analysis was used to identify potential binding pockets, and molecular docking was performed using the Glide module. Docking results were evaluated based on docking scores and interaction analysis.

### 2.8. Molecular Dynamics and Steered Molecular Dynamics

Molecular dynamics (MD) simulations were performed using Gromacs 2023.2 with the Amber14SB force field for proteins and the General Amber Force Field (GAFF) for small molecule ligands [34,35]. Water molecules were represented using the TIP3P water model [36]. The protein–ligand complex was placed in a cubic periodic water box with a minimum distance of 10 Å from the edges, and the system was neutralized with sodium (or chloride). Long-range electrostatic interactions were treated using the Particle Mesh Ewald (PME) method [37]. Energy minimization was carried out with the steepest descent algorithm for a maximum of 5000 steps to locate the energy minima. This was followed by 100 ps NVT equilibration and 100 ps NPT equilibration to stabilize the system, with production MD simulations performed at a constant temperature of 300 K and a pressure of 1 bar under periodic boundary conditions. The production run duration was 100 ns. The final frame from the MD simulation was used as the initial structure for steered molecular dynamics (SMD) simulations, conducted at 300 K and 1 bar, with a pulling rate of 0.01 nm/ps and a force constant of 1000 kJ/(mol·nm^2^). Periodic boundary conditions were maintained throughout the simulations, with a time step of 2 fs and data collection intervals of 2 ps for energy, trajectory, and structural information.

### 2.9. Statistical Analysis

All experiments, except for the preliminary screening of the 257 wild strains, were performed with at least three biological replicates. Data are presented as mean ± standard deviation. Statistical significance between wild-type and mutant phenotypes was assessed using paired *t*-tests, with significance defined as *p*-values < 0.05. No multiple comparison adjustments were applied to *p*-values.

## 3. Results

### 3.1. Expression and Characterization of TreA from E. coli

To provide a reliable source of trehalase, the *treA* gene from *E. coli*, encoding a periplasmic trehalase enzyme, was selected for expression and purification [38]. A pET28A-based inducible expression vector containing a C-terminal His-Tag was constructed using the *treA* gene sequence derived from *E. coli* DH5α, and this vector was transformed into *E. coli* BL21(DE3) cells. The successful expression and purification of the TreA protein were confirmed via SDS-PAGE analysis (Supplementary Figure S1).

The enzymatic properties of the purified TreA were systematically characterized to ensure its suitability for trehalose hydrolysis in subsequent assays. In 20 mM phosphate buffer with 10 mM trehalose as the substrate, TreA exhibited optimal activity at pH 5.5. Importantly, the enzyme maintained high activity across a broad pH range (3 to 7), indicating its robustness in various biochemical environments (Supplementary Figure S2a). Temperature profiling revealed that TreA achieves its optimal reaction rate at 37 °C, with activity remaining stable across the 35 °C to 38.5 °C range (Supplementary Figure S2b). Time-course analysis demonstrated that TreA could efficiently hydrolyze 10 mM trehalose within 30 min at an enzyme concentration of 32 μg/L, achieving a near-complete conversion rate of 98.36% (Supplementary Figure S2c). Comparative studies with commercial trehalase showed no significant difference in reaction efficiency, as both enzymes effectively hydrolyzed 10 mM trehalose (Supplementary Figure S2d). These findings established the maximum specific activity of the purified TreA as 28.125 μM/mg/min, confirming its efficacy for quantitative assays. The reliability of this method was validated by constructing a standard curve for trehalose detection using TreA (Supplementary Figure S2e). Trehalose transport activity was measured in wild-type Agt1-overexpressing and trehalose biosynthesis mutant strains (Supplementary Figure S2f). As expected, ACY285, which overexpresses Agt1, exhibited significantly enhanced trehalose transport, while the S288C derivatives with silenced *AGT1* showed low transport activity.

### 3.2. Identification and Screening of Yeast Strains with Enhanced Trehalose Transport Capacity

The trehalose transport pathway in *S. cerevisiae* plays a vital role in improving cellular stress resistance through the uptake of exogenous trehalose [18,19]. However, despite its importance, the structural and functional details of Agt1, the key transporter involved, remain largely underexplored. To address this, we screened a collection of 257 *S. cerevisiae* strains, which included two widely used laboratory strains, isolates from commercial suppliers involved in fermented food and beverage production, and wild isolates collected from vineyards, breweries, and natural habitats. This diverse collection ensured a broad spectrum of genetic diversity for the study.

**Figure 2:**
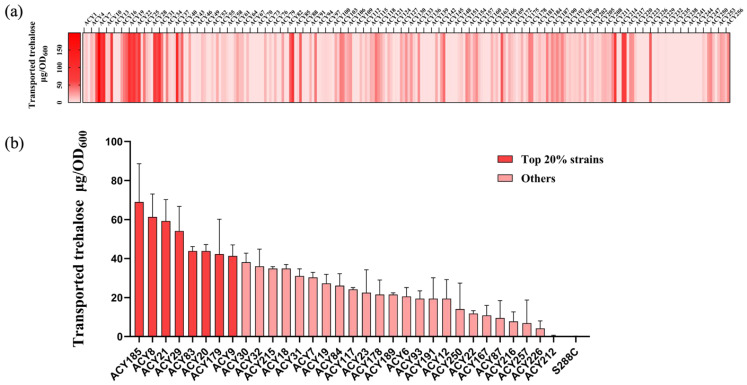
Assessment and screening of trehalose transport ability in wild *S. cerevisiae* strains. (**a**) Preliminary screening results displaying the trehalose transport capacity of all 257 wild *S. cerevisiae* strains, with strain numbers marked every three entries; (**b**) Detailed measurement and ranking of trehalose transport capacity for selected strains based on preliminary screening results. Strains with higher trehalose transport capacity (top 20%) are highlighted in darker color.

Preliminary screening revealed considerable variability in the trehalose transport capabilities of the isolates, with many strains exhibiting minimal or no capacity to transport environmental trehalose, consistent with previous reports of Agt1 silencing in *S. cerevisiae* (Figure 2a) [7]. This variability underscores the genetic diversity within the species and suggests that certain strains may possess unique genetic modifications or regulatory mechanisms that enhance trehalose uptake. To mitigate batch effects and improve accuracy, the top 10% of strains were selected for secondary screening based on their trehalose content (Figure 2b). This process identified 36 yeast strains with notably higher trehalose transport capacities (Supplementary Figure S3). Refined analysis during secondary screening pinpointed eight strains (ACY8, ACY9, ACY20, ACY21, ACY29, ACY83, ACY179, ACY185) that consistently demonstrated high transport capacity, ranking in the top 20% of the secondary screening. These results highlight the substantial variability in trehalose transport capabilities among different *S. cerevisiae* strains and suggest that the genetic diversity underlying Agt1 function could be harnessed for industrial applications requiring enhanced stress resistance.

### 3.3. Comparative Analysis of Agt1-Mediated Trehalose Transport in High-Performance Yeast Strains

Agt1-mediated cotransport of trehalose and protons remains the primary known mechanism for trehalose transmembrane transport in yeast [8,39]. To determine whether the high trehalose transport capacity observed in the eight screened yeast strains was due to Agt1, we constructed constitutive Agt1 overexpression strains in the S288C lab strain background, which lacks native *AGT1* expression [15]. This setup ensured that observed differences in trehalose transport were due solely to the overexpressed *AGT1* variants.

**Figure 3:**
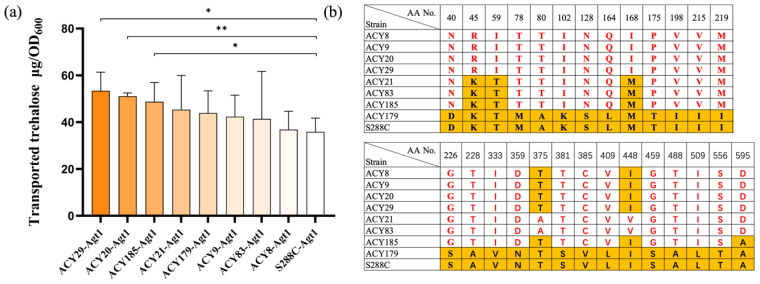
Efficiency determination and sequence analysis of Agt1 in high trehalose-transporting strains. (**a**) Trehalose transport capability of Agt1 from different strains with high trehalose transport ability, expressed in an S288C lab strain background. Significant differences between groups are indicated by asterisks (*, *p* < 0.05; **, *p* < 0.01). (**b**) Sequence differences in Agt1 from 8 strains identified as having the highest trehalose transport ability. The amino acid residues with differences are marked in red. The orange background indicates strains with amino acid sequences identical to those in the S288C genetic background.

Trehalose transport assays revealed variability in transport capacity across the strains, including the S288C control strain expressing its own *AGT1* gene (Figure 3a). Overexpression of *AGT1* from strains such as ACY20, ACY29, and ACY185 resulted in significantly higher transport capacity compared to the control, with ACY29 showing the highest activity at 1.49 times greater than S288C (Figure 3a). Interestingly, while the ACY185 strain itself exhibited slightly higher trehalose uptake efficiency, the overexpression of its *AGT1* in the S288C background showed slightly lower efficiency. Additionally, despite the native low trehalose uptake of S288C (Figure 2b), overexpressing its own Agt1 protein improved transport efficiency (Figure 3a), highlighting that Agt1 activity may be regulated by factors beyond its protein structure. These findings suggest that, while the Agt1 protein structure is critical for substrate transport, factors such as expression levels, membrane localization, or post-translational modifications may significantly influence Agt1 activity [40,41,42]. Post-translational modifications or changes in Agt1 expression could explain why some strains, despite having identical Agt1 sequences, exhibit different transport capacities. This highlights the importance of considering Agt1 regulatory mechanisms in future studies to develop strains with enhanced trehalose uptake efficiency, which could be beneficial in industrial applications.

Sequencing and alignment of the *AGT1* genes from the nine yeast strains revealed that ACY8, ACY9, ACY20, and ACY29 share identical Agt1 sequences, while ACY179 is identical to S288C (Supplementary Table S4). ACY21 and ACY83 are identical to each other, and ACY185 has a unique sequence (Figure 3b). The identical Agt1 protein sequences in ACY8, ACY9, ACY20, and ACY29 further suggest factors beyond the primary sequence—such as post-translational modifications or expression levels, likely contribute to the observed variability in trehalose transport capacity [40,41,42]. The unique Agt1 sequences identified in ACY21 and ACY185 represent novel variants, offering new insights into Agt1 structure–function relationships.

### 3.4. Residue Interactions and Transport Dynamics of Trehalose in Agt1

**Figure 4:**
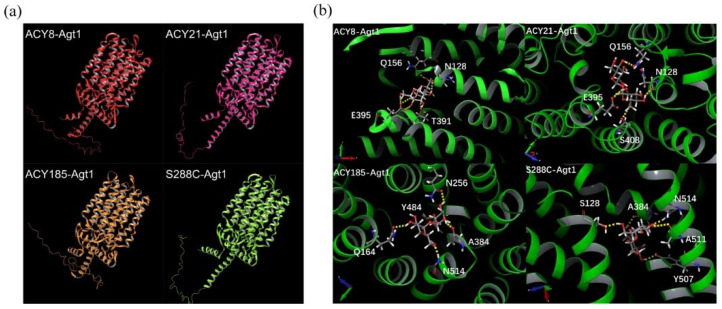
Structural modeling and trehalose docking in Agt1 variants. (**a**) Structural models of Agt1 from ACY8, ACY21, ACY185, and S288C generated using ColabFold 15.5; (**b**) Trehalose docking in Agt1 variants, highlighting key amino acids involved in hydrogen bond formation with trehalose. The trehalose molecule is shown surrounded by stick models representing the interacting amino acids, with hydrogen bonds depicted as yellow dashed lines. The orientation of each protein structure is indicated by the coordinate axes in the lower-left corner.

To further investigate the mechanism of Agt1-mediated trehalose transport, four Agt1 protein sequences—from ACY8, ACY21, ACY185, and S288C—were selected and modeled using the ColabFold online tool (Figure 4a). A comparison of these models revealed that each Agt1 structure contained 23 α-helices, with helices TM3-10 and TM13-21 located in the transmembrane region. Smaller helices (TM3, TM4; TM9, TM10; TM13, TM14, TM15; TM15, TM18) symmetrically formed structures similar to larger transmembrane α-helices. The overall structures of the four proteins were highly similar, with only slight variations in channel width (Supplementary Table S5). These findings suggest that differences in trehalose transport efficiency due to mutations are likely not caused by changes in the spatial structure, but rather by alterations in the potential energy of key residues involved in trehalose transport.

To test this hypothesis, molecular docking was performed using Schrödinger2021-4 software to identify the optimal trehalose binding sites within the Agt1 transmembrane channel (Figure 4b). The docking analysis revealed a hydrophilic cavity whereas dense hydrogen bond network was formed with several key transmembrane residues. Despite strong trehalose binding affinity observed across all four Agt1 structures, differences emerged in the specific binding sites and hydrogen bond networks. Agt1 from ACY185 and S288C shared similar substrate binding positions, with both forming hydrogen bonds at N514 and A384. However, their remaining bonding sites varied. In ACY185, the hydrogen bond network was more evenly distributed across different helices (Q164 Y484, and N256), while in S288C, except for S128, the bonding sites were predominantly located on one side of the channel. Agt1 from ACY8 and ACY21 displayed very similar hydrogen bond networks, differing by only one binding site in close spatial proximity.

The docking analysis also showed that the best trehalose binding sites in ACY185 and S288C were located closer to the middle or bottom of the channel, while in ACY8 and ACY21, they were nearer to the channel entrance. The presence of many hydrophilic side chains within the Agt1 channel suggests that trehalose can bind at multiple sites. While a shift in the best binding site location does not necessarily indicate a significant change in the transport mechanism, the high binding affinity at these sites suggests that residues involved in forming hydrogen bonds with trehalose could play crucial roles in the transport process. Several residues, including S128, Q156, A384, E395, and N514, were frequently involved in hydrogen bond formation across multiple Agt1 variants, indicating their potential importance for trehalose binding and transport. Conversely, residues such as Q164, N256, T391, S408, Y484, Y507, and A511 appear less consistently in the best docking results, suggesting their roles might be more context-dependent or replaceable in the transport mechanism.

### 3.5. Key Residue Interactions in Agt1-Mediated Trehalose Transport

**Figure 5:**
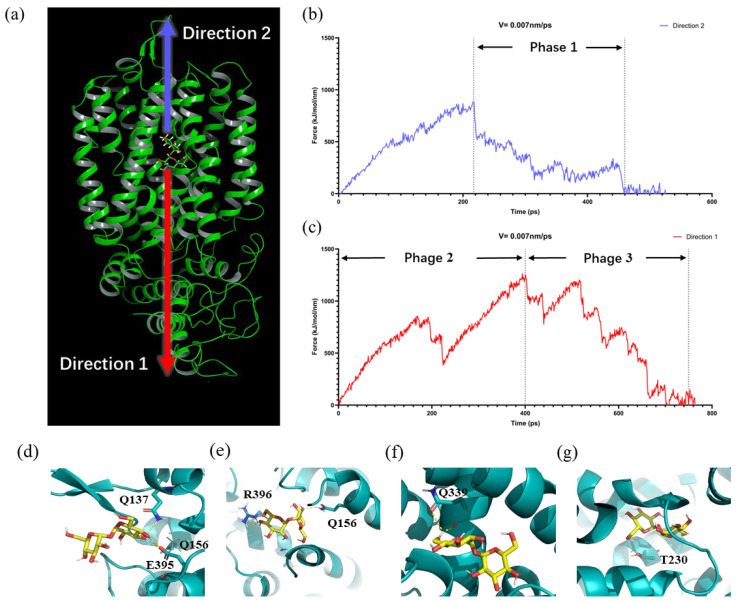
Steered molecular dynamics analysis of trehalose in Agt1. (**a**) Schematic representation of the direction of force applied to the trehalose molecule during steered molecular dynamics (SMD). Direction 1 corresponds to the transmembrane movement of trehalose, while Direction 2 is the opposite; (**b**,**c**) Force curves for trehalose at a pulling speed of 0.007 nm/ps. Force peaks mark distinct stages (Stage 1, Stage 2, Stage 3) as trehalose moves through the Agt1 channel; (**d**,**e**) Hydrogen bonds formed during Stage 1, where residues stabilize trehalose entry into the channel; (**f**) Hydrogen bonds in Stage 2, where trehalose encounters and overcomes significant resistance; (**g**) Hydrogen bonds in Stage 3, facilitating trehalose exit from the channel.

Previous studies have explained the mechanisms of hexose and disaccharide transporters through hydrogen bonding between amino acid residues within the transporter channel and the substrate [43,44]. However, while substrate docking has identified key residues in Agt1 that closely interact with the substrate during transport, it does not fully explain how trehalose is influenced by Agt1 as it moves through the channel. To address this, steered molecular dynamics (SMD) simulations were employed, using the docking results of S288C-Agt1 with trehalose to simulate its transport process within the Agt1 channel (Figure 4b). The force profiles from the SMD simulations divide trehalose movement within Agt1 into three distinct phases (Figure 5a–c). In the first phase (200–460 ps, direction 2), trehalose is attracted to the entrance of Agt1 and initially interacts with channel residues, reflecting the process of its capture and entry into the channel (Figure 5b). The second phase (0–400 ps, direction 1) involves trehalose moving from the lowest energy binding site within the channel toward the channel exit. The third phase (400–750 ps, direction 1) involves trehalose overcoming steric hindrance at the exit of the channel and ultimately leaving the channel (Figure 5c).

During these phases, peaks in the force profiles indicate points of maximum resistance encountered by trehalose, which are critical for understanding its transport mechanism. The frame-by-frame analysis identified key conformations where trehalose forms hydrogen bonds with Agt1 residues (Figure 5d–g). In Phase 1, residues 137, 256, 395, and 396 form hydrogen bonds with trehalose, influencing its position and conformation (Figure 5d,e). In Phase 2, residue Q339 forms a hydrogen bond with trehalose at the point of maximum resistance, suggesting that steric hindrance at this site is crucial for selective transport and may be related to conformational changes in the channel (Supplementary Figure S4). In Phase 3, residue T230 forms a hydrogen bond with trehalose, with the observed resistance likely due to similar steric effects as in Phase 2. This hydrogen bond may facilitate trehalose passage through the channel (Figure 5g).

### 3.6. Validation of Key Residues in Agt1-Mediated Trehalose Transport

To investigate the roles of key residues identified through substrate docking and SMD on Agt1-mediated transport trehalose, single-point mutations were introduced into S288C-Agt1 based on computational predictions. Alanine was chosen due to its minimal side chain, making it ideal for assessing the functional contributions of the original residues. The selected residues included those in hydrogen bonding in docking studies and those affecting trehalose movement in SMD simulations (Table 1).

**Figure 6:**
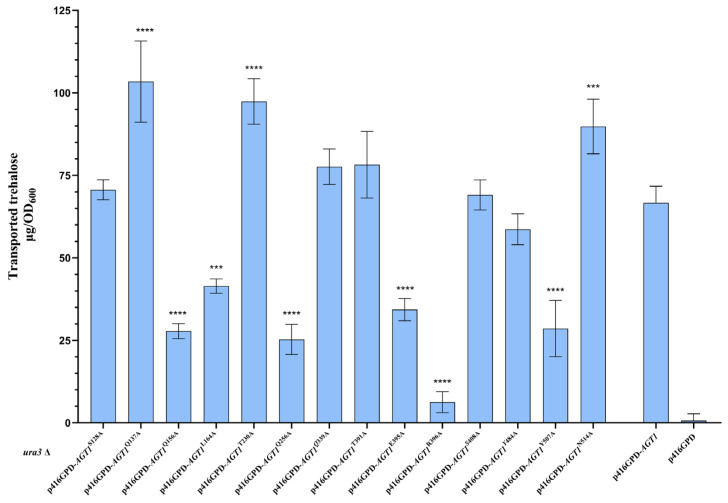
Trehalose transport activity of Agt1p mutants. This bar graph compares the trehalose transport activity of Agt1 mutants to the wild type. The positive control is the S288C strain overexpressing Agt1, and the negative control is the S288C strain with the empty vector p416GPD. Data represent the mean ± SD from three replicates. Asterisks indicate significance: *** *p* < 0.001, **** *p* < 0.0001.

Trehalose transport assays in S288C strains expressing these Agt1 mutants revealed notable changes in transport efficiency (Figure 6). Mutants Q137A, T230A, and N514A showed increased transport efficiency, while Q156A, L164A, Q256A, E395A, R396A, and Y507A exhibited reduced functionality, suggesting the importance of these residues in trehalose transport. The increased efficiency observed in the Q137A mutant suggests that Q137 may play a role in attracting and capturing trehalose or other α-glucosides. However, at high trehalose concentrations, this strong affinity could impede trehalose entry into the channel, reducing overall transport efficiency. Alanine mutations at residues E395, R396, and Q156, which are located near the channel entrance, result in decreased transport efficiency, indicating their crucial role in trehalose recognition and stabilization (Supplementary Figure S4). Furthermore, residues Q256, Y507, and N514, situated near the base on the channel and the optimal binding site, were found to be critical for efficient transport. Q256, part of the hydrogen bond network, likely contributes to the stabilization of trehalose during transport. Y507 and N514 are located in a hydrophobic region with significant steric hindrance, where trehalose undergoes prolonged retention and conformational changes (Supplementary Figure S4). The spatial structure, charge distribution, and hydrogen bond network in this region are essential for proper trehalose positioning and conformational shifts, with mutations notably impacting transport efficiency. Although Y507 and N514 maintain similar spatial positions, their transport activity differs. Y507 forms a concave structure toward the channel, important for adjusting the trehalose conformation during transport. Mutation of alanine, which has a smaller side chain and is hydrophobic, disrupts this structure and reduces trehalose transport efficiency. In contrast, N514 is convex and located on the outer surface of the channel. Mutation of alanine enhances this spatial characteristic, potentially improving trehalose structural changes and transport efficiency at this site. T230, located within the core of Agt1, is implicated in Stage 3 of trehalose transport (Figure 5b and Supplementary Figure S4). Its mutation led to decreased transport activity, suggesting its involvement in facilitating trehalose passage. The alanine mutation at this site also reduced steric hindrance, potentially enhancing transport efficiency.

## 4. Discussion

Trehalose is a well-known stress protectant, widely recognized for its protective effects against various environmental stresses in both yeast and other organisms [19,20,45,46]. Numerous studies have extensively investigated the trehalose metabolic pathway in *S. cerevisiae* and its regulatory mechanisms [5,7,47]. However, recent evidence suggests that the protective effects of intracellular trehalose are selective, providing protection against certain stressors but not others [7]. To directly assess the physiological role of trehalose, we used the Agt1 transporter as a tool, utilizing a constitutively expressed version of *AGT1* in combination with trehalose metabolism mutants. This approach allows for the direct regulation of intracellular trehalose levels without altering other metabolic pathways. Using this approach, we demonstrated that while trehalose cannot provide heat tolerance, it effectively protects yeast against desiccation and freeze-thaw stress [19,20]. These findings suggest that the protective effects of trehalose may be most effective under conditions of low water activity, such as during desiccation or freezing.

Agt1 is a member of the *MAL* family of α-glucoside transporters, distinguished by its broad substrate specificity, particularly its high efficiency in transporting trehalose. In addition to trehalose, Agt1 can also facilitate the transport of other sugars, including sucrose and maltotriose [16,17,48,49]. Previous studies have leveraged Agt1 expression to improve the uptake of maltotriose from grapes and sucrose from sugarcane during brewing processes [50,51]. However, despite its broad transport capabilities, research on Agt1 has primarily focused on its role in transporting maltose, sucrose, and maltotriose, with less attention given to its trehalose transport function. Key residues such as Glu-120, Asp-123, Glu-167, and Arg-504 have been identified as critical for the transport of various substrates by Agt1 [44]. Substitution of these residues with alanine leads to a loss or reduction of transport function, highlighting their crucial roles in Agt1 activity. Thr-505, located after the TM11 and TM12 transmembrane helices of Agt1, has been shown to be important for maltotriose transport; mutation of this residue to isoleucine enhances maltotriose transport [43]. Despite these insights into the broader substrate transport capabilities of Agt1, there has been relatively little research specifically focused on understanding its role in trehalose transport.

In our study, we aimed to identify *S. cerevisiae* strains with high trehalose transport efficiency by screening a large number of wild strains. For the first time, both molecular docking and SMD simulations were employed to investigate amino acid residues that may impact the function of Agt1 in trehalose transport. Guided by these computational simulations, nine residues were identified as playing crucial roles in this process. While some uncertainties remain—such as the variability in experimental validation for residues identified through substrate docking—our study provides valuable insights into the transport mechanism of Agt1. Specifically, the SMD method demonstrated substantial potential for advancing the understanding of transport mechanisms not only in Agt1 but also in other α-glucoside, disaccharide, and hexose transport proteins. One notable finding was that while certain residues identified through substrate docking did not consistently align with experimental results as accurately as those identified through SMD, this discrepancy may be attributed to the presence of polar amino acids within the Agt1 channel. These polar residues can form a complex hydrogen bond network with trehalose during transport. When certain residues are mutated, the potential loss of function may be compensated by nearby polar amino acids, leading to no significant difference in trehalose transport efficiency during experimental measurements. Nevertheless, the identification of five residues with a 55% accuracy compared to computational predictions underscores the value of this approach.

Notably, the head region of the Agt1 channel exhibited a strong affinity for trehalose in our computational simulations. This region is rich in amino acids capable of forming hydrogen bonds with trehalose, exhibits a strongly positive surface charge, and includes a rare negatively charged pocket attributed to residue E395. We hypothesize that this region is critical for the binding, positioning, and conformational adjustment of trehalose during Agt1-mediated transport. Mutation analysis partially corroborated this hypothesis. The point mutation at residue R396 almost completely abolished the trehalose transport activity of Agt1, while mutations at Q156 and E395 significantly reduced the transport rate. Although the mutation at Q137 objectively enhanced Agt1 transport efficiency, it is important to note that Q137 is located at the outermost part of the Agt1 head region, where it exerts a strong attractive force on trehalose. Under our experimental conditions, where the extracellular trehalose concentration was relatively high, the importance of Agt1 affinity for trehalose in facilitating substrate capture was greatly diminished. Instead, this strong affinity might have slowed down the translocation rate of individual trehalose molecules. Further investigation involving a wider range of trehalose concentrations, a more diverse set of α-glucosides, and an expanded combination of point mutations could provide deeper insights into whether this region is indeed crucial for substrate recognition and capture by Agt1.

By integrating modern AI-based tools such as AlphaFold2 and Schrödinger2021-4, we were able to extend previous insights provided by studies and push the boundaries of our understanding of Agt1 substrate specificity [44]. The use of these advanced computational tools enabled us to not only validate previously known residues but also to identify novel interactions critical for trehalose transport. This combination of molecular dynamics simulations and experimental mutation analyses allows for a more comprehensive understanding of the Agt1 transport mechanism, contributing valuable insights for potential industrial applications that focus on improving yeast strains for stress resistance.

## Figures and Tables

**Table 1:** Key amino acid residues in Agt1 involved in trehalose docking and transport dynamics.

Agt1 Mutation Site	Function
S128A	Forms hydrogen bonds in ACY8, ACY21, S288C trehalose docking sites; key position in stretching dynamics.
Q137A	Key position in stretching dynamics, forms hydrogen bonds with trehalose.
Q156A	Forms hydrogen bonds in ACY8, ACY21 docking sites; key position in stretching dynamics.
L164A	Forms hydrogen bonds in ACY185 docking site.
T230A	Key position in stretching dynamics, forms hydrogen bonds with trehalose.
Q256A	Forms hydrogen bonds in ACY185 docking site.
Q339A	Key position in stretching dynamics, forms hydrogen bonds with trehalose.
T391A	Forms hydrogen bonds in ACY8 docking site.
E395A	Forms hydrogen bonds in ACY8, ACY21 docking sites; key position in stretching dynamics.
R396A	Key position in stretching dynamics, forms hydrogen bonds with trehalose.
S408A	Forms hydrogen bonds in ACY21 docking site.
Y484A	Forms hydrogen bonds in ACY185 docking site.
Y507A	Forms hydrogen bonds in S288C docking site.
N514A	Forms hydrogen bonds in S288C, ACY185 docking site.

## Data Availability

The original contributions presented in the study are included in the article/Supplementary Materials, further inquiries can be directed to the corresponding author/s.

## Supplementary Materials

The following supporting information can be downloaded at: https://www.mdpi.com/article/10.3390/jof10110781/s1, Figure S1: TreA trehalase expression and purification; Figure S2: Comprehensive characterization and functional validation of TreA enzyme from *E. coli* in trehalose hydrolysis; Figure S3: Ranking and selection of initial screened strains based on trehalose transport activity; Figure S4: Key residues influencing trehalose transport and their surface electrostatic potential; Table S1: Strains used in this study; Table S2: Plasmids used in this study; Table S3: Primers used in this study; Table S4: Agt 1 sequence in yeast strains with high trehalose transport capability; Table S5: Root-mean-square deviation (RMSD) comparison of Alphafold-simulated protein structures.

## References

[1] Chen A., Tapia H., Goddard J.M., Gibney P.A. (2022). Trehalose and Its Applications in the Food Industry. Compr. Rev. Food Sci. Food Saf..

[2] Elbein A.D., Pan Y.T., Pastuszak I., Carroll D. (2003). New Insights on Trehalose: A Multifunctional Molecule. Glycobiology.

[3] Jain N.K., Roy I. (2009). Effect of Trehalose on Protein Structure. Protein Sci..

[4] François J., Parrou J.L. (2001). Reserve Carbohydrates Metabolism in the Yeast *Saccharomyces cerevisiae*. FEMS Microbiol. Rev..

[5] Chen A., Vargas-Smith J., Tapia H., Gibney P.A. (2022). Characterizing Phenotypic Diversity of Trehalose Biosynthesis Mutants in Multiple Wild Strains of *Saccharomyces cerevisiae*. G3 Genes|Genomes|Genet..

[6] Bisson L.F., Fan Q., Walker G.A. (2016). Sugar and Glycerol Transport in *Saccharomyces cerevisiae*. Adv. Exp. Med. Biol..

[7] Gibney P.A., Schieler A., Chen J.C., Rabinowitz J.D., Botstein D. (2015). Characterizing the in Vivo Role of Trehalose in *Saccharomyces cerevisiae* Using the AGT1 Transporter. Proc. Natl. Acad. Sci. USA.

[8] Vidgren V., Londesborough J. (2012). Characterization of the Saccharomycesbayanus-Type Agt1 Transporter of Lager Yeast. J. Inst. Brew..

[9] Stambuk B.U., Alves S.L., Hollatz C., Zastrow C.R. (2006). Improvement of Maltotriose Fermentation by *Saccharomyces cerevisiae*. Lett. Appl. Microbiol..

[10] Han E.K., Cotty F., Sottas C., Jiang H., Michels C.A. (1995). Characterization of AGT1 Encoding a General A-glucoside Transporter from Saccharomyces. Mol. Microbiol..

[11] Vidgren V., Kankainen M., Londesborough J., Ruohonen L. (2011). Identification of Regulatory Elements in the AGT1 Promoter of Ale and Lager Strains of Brewer’s Yeast. Yeast.

[12] Rodrigues C.I.S., Wahl A., Gombert A.K. (2021). Aerobic Growth Physiology of *Saccharomyces cerevisiae* on Sucrose Is Strain-Dependent. FEMS Yeast Res..

[13] Vidgren V., Ruohonen L., Londesborough J. (2005). Characterization and Functional Analysis of the MAL and MPH Loci for Maltose Utilization in Some Ale and Lager Yeast Strains. Appl. Environ. Microbiol..

[14] Vidgren V., Multanen J.P., Ruohonen L., Londesborough J. (2010). The Temperature Dependence of Maltose Transport in Ale and Lager Strains of Brewer’s Yeast. FEMS Yeast Res..

[15] Bell P.J.L., Higgins V.J., Dawes I.W., Bissinger P.H. (1997). Tandemly Repeated 147 Bp Elements Cause Structural and Functional Variation in Divergent MAL Promoters of *Saccharomyces cerevisiae*. Yeast.

[16] Stambuk B.U., De Araujo P.S. (2001). Kinetics of Active α-Glucoside Transport in *Saccharomyces cerevisiae*. FEMS Yeast Res..

[17] Plourde-Owobi L., Durner S., Goma G., François J. (2000). Trehalose Reserve in *Saccharomyces cerevisiae*: Phenomenon of Transport, Accumulation and Role in Cell Viability. Int. J. Food Microbiol..

[18] Kulikova-Borovikova D., Lisi S., Dauss E., Alamae T., Buzzini P., Hallsworth J.E., Rapoport A. (2018). Activity of the α-Glucoside Transporter Agt1 in *Saccharomyces cerevisiae* Cells during Dehydration-Rehydration Events. Fungal Biol..

[19] Chen A., Gibney P.A. (2022). Intracellular Trehalose Accumulation via the Agt1 Transporter Promotes Freeze–Thaw Tolerance in *Saccharomyces cerevisiae*. J. Appl. Microbiol..

[20] Tapia H., Young L., Fox D., Bertozzi C.R., Koshland D. (2015). Increasing Intracellular Trehalose Is Sufficient to Confer Desiccation Tolerance to *Saccharomyces cerevisiae*. Proc. Natl. Acad. Sci. USA.

[21] Mirdita M., Schütze K., Moriwaki Y., Heo L., Ovchinnikov S., Steinegger M. (2022). ColabFold: Making Protein Folding Accessible to All. Nat. Methods.

[22] Jumper J., Evans R., Pritzel A., Green T., Figurnov M., Ronneberger O., Tunyasuvunakool K., Bates R., Žídek A., Potapenko A. (2021). Highly Accurate Protein Structure Prediction with AlphaFold. Nature.

[23] Friesner R.A., Banks J.L., Murphy R.B., Halgren T.A., Klicic J.J., Mainz D.T., Repasky M.P., Knoll E.H., Shelley M., Perry J.K. (2004). Glide: A New Approach for Rapid, Accurate Docking and Scoring. 1. Method and Assessment of Docking Accuracy. J. Med. Chem..

[24] Halgren T.A., Murphy R.B., Friesner R.A., Beard H.S., Frye L.L., Pollard W.T., Banks J.L. (2004). Glide: A New Approach for Rapid, Accurate Docking and Scoring. 2. Enrichment Factors in Database Screening. J. Med. Chem..

[25] Yang Y., Yao K., Repasky M.P., Leswing K., Abel R., Shoichet B.K., Jerome S.V. (2021). Efficient Exploration of Chemical Space with Docking and Deep Learning. J. Chem. Theory Comput..

[26] Friesner R.A., Murphy R.B., Repasky M.P., Frye L.L., Greenwood J.R., Halgren T.A., Sanschagrin P.C., Mainz D.T. (2006). Extra Precision Glide: Docking and Scoring Incorporating a Model of Hydrophobic Enclosure for Protein-Ligand Complexes. J. Med. Chem..

[27] Van Der Spoel D., Lindahl E., Hess B., Groenhof G., Mark A.E., Berendsen H.J.C. (2005). GROMACS: Fast, Flexible, and Free. J. Comput. Chem..

[28] Guthrie C., Fink G.R. (1991). Guide to Yeast Genetics and Molecular Biology. Methods in Enzymology.

[29] Sikorski R.S., Hieter P. (1989). A System of Shuttle Vectors and Yeast Host Strains Designed for Efficient Manipulation of DNA in *Saccharomyces cerevisiae*. Genetics.

[30] Mumberg D., Müller R., Funk M. (1995). Yeast Vectors for the Controlled Expression of Heterologous Proteins in Different Genetic Backgrounds. Gene.

[31] Wong E.D., Miyasato S.R., Aleksander S., Karra K., Nash R.S., Skrzypek M.S., Weng S., Engel S.R., Cherry J.M. (2023). Saccharomyces Genome Database Update: Server Architecture, Pan-Genome Nomenclature, and External Resources. Genetics.

[32] Kumar S., Stecher G., Li M., Knyaz C., Tamura K. (2018). MEGA X: Molecular Evolutionary Genetics Analysis across Computing Platforms. Mol. Biol. Evol..

[33] Edgar R.C. (2004). MUSCLE: Multiple Sequence Alignment with High Accuracy and High Throughput. Nucleic Acids Res..

[34] Wang J., Wolf R.M., Caldwell J.W., Kollman P.A., Case D.A. (2004). Development and Testing of a General Amber Force Field. J. Comput. Chem..

[35] Wang J., Wang W., Kollman P.A., Case D.A. (2006). Automatic Atom Type and Bond Type Perception in Molecular Mechanical Calculations. J. Mol. Graph. Model..

[36] MacKerell A.D., Bashford D., Bellott M., Dunbrack R.L., Evanseck J.D., Field M.J., Fischer S., Gao J., Guo H., Ha S. (1998). All-Atom Empirical Potential for Molecular Modeling and Dynamics Studies of Proteins. J. Phys. Chem. B.

[37] Darden T., York D., Pedersen L. (1993). Particle Mesh Ewald: An N⋅log(N) Method for Ewald Sums in Large Systems. J. Chem. Phys..

[38] Strom A.R., Kaasen I. (1993). Trehalose Metabolism in Escherichia Coli: Stress Protection and Stress Regulation of Gene Expression. Mol. Microbiol..

[39] Stambuk B.U., Panek A.D., Crowe J.H., Crowe L.M., De Araujo P.S. (1998). Expression of High-Affinity Trehalose-H+ Symport in *Saccharomyces cerevisiae*. Biochim. Biophys. Acta—Gen. Subj..

[40] Tripodi F., Nicastro R., Reghellin V., Coccetti P. (2015). Post-translational modifications on yeast carbon metabolism: Regulatory mechanisms beyond transcriptional control. Biochim. Biophys. Acta.

[41] Vidgren V., Londesborough J. (2018). Overexpressed maltose transporters in laboratory and lager yeasts: Localization and competition with endogenous transporters. Yeast.

[42] Izawa S., Inoue Y. (2009). Post-transcriptional regulation of gene expression in yeast under ethanol stress. Biotechnol. Appl. Biochem..

[43] Smit A., Moses S.G., Pretorius I.S., Cordero Otero R.R. (2008). The Thr505 and Ser557 Residues of the AGT1-Encoded α-Glucoside Transporter Are Critical for Maltotriose Transport in *Saccharomyces cerevisiae*. J. Appl. Microbiol..

[44] Trichez D., Knychala M.M., Figueiredo C.M., Alves S.L., da Silva M.A., Miletti L.C., de Araujo P.S., Stambuk B.U. (2019). Key Amino Acid Residues of the AGT1 Permease Required for Maltotriose Consumption and Fermentation by *Saccharomyces cerevisiae*. J. Appl. Microbiol..

[45] Sebollela A., Louzada P.R., Sola-Penna M., Sarone-Williams V., Coelho-Sampaio T., Ferreira S.T. (2004). Inhibition of Yeast Glutathione Reductase by Trehalose: Possible Implications in Yeast Survival and Recovery from Stress. Int. J. Biochem. Cell Biol..

[46] Magalhães R.S.S., Popova B., Braus G.H., Outeiro T.F., Eleutherio E.C.A. (2018). The Trehalose Protective Mechanism during Thermal Stress in *Saccharomyces cerevisiae*: The Roles of Ath1 and Agt1. FEMS Yeast Res..

[47] Jules M., Guillou V., François J., Parrou J.L. (2004). Two Distinct Pathways for Trehalose Assimilation in the Yeast *Saccharomyces cerevisiae*. Appl. Environ. Microbiol..

[48] Stambuk B.U., Batista A.S., De Araujo P.S. (2000). Kinetics of Active Sucrose Transport in *Saccharomyces cerevisiae*. J. Biosci. Bioeng..

[49] Teste M.A., Marie François J., Parrou J.L. (2010). Characterization of a New Multigene Family Encoding Isomaltases in the Yeast *Saccharomyces cerevisiae*, the IMA Family. J. Biol. Chem..

[50] Muller G., de Godoy V.R., Dário M.G., Duval E.H., Alves-Jr S.L., Bücker A., Rosa C.A., Dunn B., Sherlock G., Stambuk B.U. (2023). Improved Sugarcane-Based Fermentation Processes by an Industrial Fuel-Ethanol Yeast Strain. J. Fungi.

[51] Cousseau F.E.M., Alves S.L., Trichez D., Stambuk B.U. (2013). Characterization of Maltotriose Transporters from the *Saccharomyces eubayanus* Subgenome of the Hybrid *Saccharomyces pastorianus* Lager Brewing Yeast Strain Weihenstephan 34/70. Lett. Appl. Microbiol..

