# Supplementary material for: Key Amino Acid Residues of the Agt1 Transporter for Trehalose Transport by Saccharomyces cerevisiae
Source: J Fungi (Basel). 2024 Nov 11;10(11):781. doi: 10.3390/jof10110781 (PMC11595304; doi:10.3390/jof10110781)
Supplement: Supplementary file 1 [file jof-10-00781-s001.zip › jof-3265303-supplementary.pptx]

## Slide 1
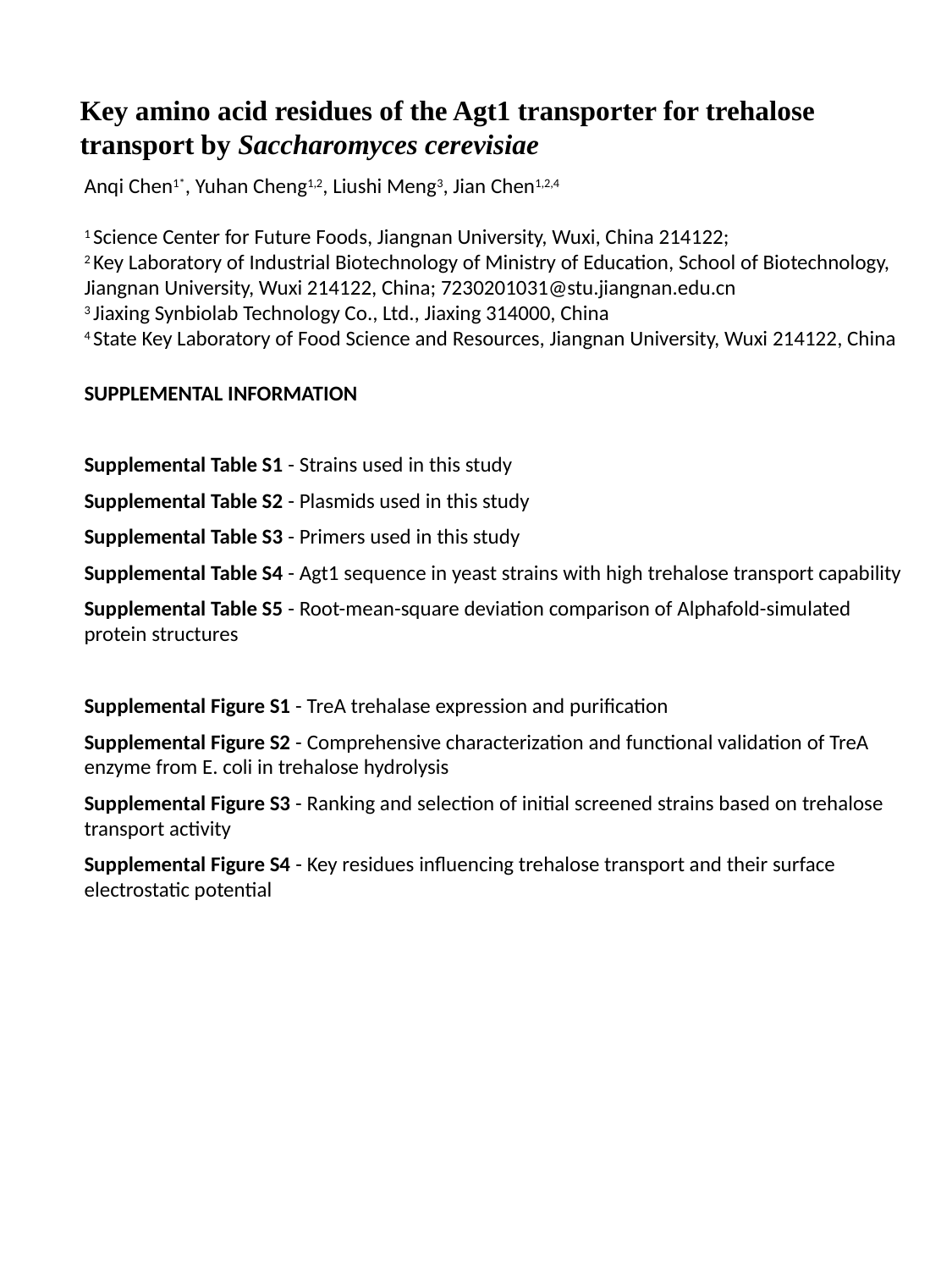

Key amino acid residues of the Agt1 transporter for trehalose transport by Saccharomyces cerevisiae
Anqi Chen1*, Yuhan Cheng1,2, Liushi Meng3, Jian Chen1,2,4
1 Science Center for Future Foods, Jiangnan University, Wuxi, China 214122;
2 Key Laboratory of Industrial Biotechnology of Ministry of Education, School of Biotechnology, Jiangnan University, Wuxi 214122, China; 7230201031@stu.jiangnan.edu.cn
3 Jiaxing Synbiolab Technology Co., Ltd., Jiaxing 314000, China
4 State Key Laboratory of Food Science and Resources, Jiangnan University, Wuxi 214122, China
SUPPLEMENTAL INFORMATION
Supplemental Table S1 - Strains used in this study
Supplemental Table S2 - Plasmids used in this study
Supplemental Table S3 - Primers used in this study
Supplemental Table S4 - Agt1 sequence in yeast strains with high trehalose transport capability
Supplemental Table S5 - Root-mean-square deviation comparison of Alphafold-simulated protein structures
Supplemental Figure S1 - TreA trehalase expression and purification
Supplemental Figure S2 - Comprehensive characterization and functional validation of TreA enzyme from E. coli in trehalose hydrolysis
Supplemental Figure S3 - Ranking and selection of initial screened strains based on trehalose transport activity
Supplemental Figure S4 - Key residues influencing trehalose transport and their surface electrostatic potential

## Slide 2
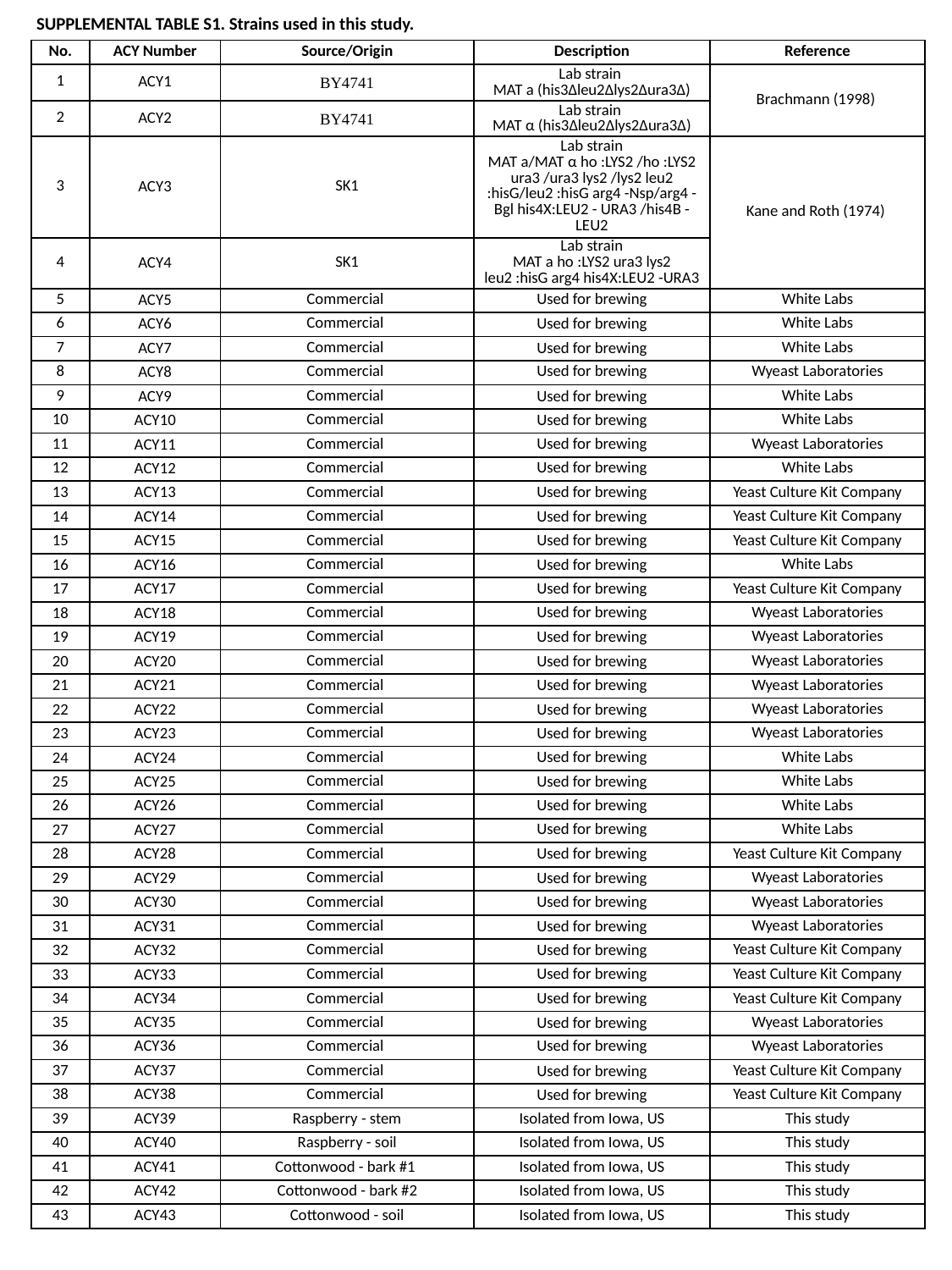

SUPPLEMENTAL TABLE S1. Strains used in this study.
| No. | ACY Number | Source/Origin | Description | Reference |
| --- | --- | --- | --- | --- |
| 1 | ACY1 | BY4741 | Lab strain MAT a (his3Δleu2Δlys2Δura3Δ) | Brachmann (1998) |
| 2 | ACY2 | BY4741 | Lab strain MAT α (his3Δleu2Δlys2Δura3Δ) | |
| 3 | ACY3 | SK1 | Lab strain MAT a/MAT α ho :LYS2 /ho :LYS2 ura3 /ura3 lys2 /lys2 leu2 :hisG/leu2 :hisG arg4 -Nsp/arg4 -Bgl his4X:LEU2 - URA3 /his4B -LEU2 | Kane and Roth (1974) |
| 4 | ACY4 | SK1 | Lab strain MAT a ho :LYS2 ura3 lys2 leu2 :hisG arg4 his4X:LEU2 -URA3 | |
| 5 | ACY5 | Commercial | Used for brewing | White Labs |
| 6 | ACY6 | Commercial | Used for brewing | White Labs |
| 7 | ACY7 | Commercial | Used for brewing | White Labs |
| 8 | ACY8 | Commercial | Used for brewing | Wyeast Laboratories |
| 9 | ACY9 | Commercial | Used for brewing | White Labs |
| 10 | ACY10 | Commercial | Used for brewing | White Labs |
| 11 | ACY11 | Commercial | Used for brewing | Wyeast Laboratories |
| 12 | ACY12 | Commercial | Used for brewing | White Labs |
| 13 | ACY13 | Commercial | Used for brewing | Yeast Culture Kit Company |
| 14 | ACY14 | Commercial | Used for brewing | Yeast Culture Kit Company |
| 15 | ACY15 | Commercial | Used for brewing | Yeast Culture Kit Company |
| 16 | ACY16 | Commercial | Used for brewing | White Labs |
| 17 | ACY17 | Commercial | Used for brewing | Yeast Culture Kit Company |
| 18 | ACY18 | Commercial | Used for brewing | Wyeast Laboratories |
| 19 | ACY19 | Commercial | Used for brewing | Wyeast Laboratories |
| 20 | ACY20 | Commercial | Used for brewing | Wyeast Laboratories |
| 21 | ACY21 | Commercial | Used for brewing | Wyeast Laboratories |
| 22 | ACY22 | Commercial | Used for brewing | Wyeast Laboratories |
| 23 | ACY23 | Commercial | Used for brewing | Wyeast Laboratories |
| 24 | ACY24 | Commercial | Used for brewing | White Labs |
| 25 | ACY25 | Commercial | Used for brewing | White Labs |
| 26 | ACY26 | Commercial | Used for brewing | White Labs |
| 27 | ACY27 | Commercial | Used for brewing | White Labs |
| 28 | ACY28 | Commercial | Used for brewing | Yeast Culture Kit Company |
| 29 | ACY29 | Commercial | Used for brewing | Wyeast Laboratories |
| 30 | ACY30 | Commercial | Used for brewing | Wyeast Laboratories |
| 31 | ACY31 | Commercial | Used for brewing | Wyeast Laboratories |
| 32 | ACY32 | Commercial | Used for brewing | Yeast Culture Kit Company |
| 33 | ACY33 | Commercial | Used for brewing | Yeast Culture Kit Company |
| 34 | ACY34 | Commercial | Used for brewing | Yeast Culture Kit Company |
| 35 | ACY35 | Commercial | Used for brewing | Wyeast Laboratories |
| 36 | ACY36 | Commercial | Used for brewing | Wyeast Laboratories |
| 37 | ACY37 | Commercial | Used for brewing | Yeast Culture Kit Company |
| 38 | ACY38 | Commercial | Used for brewing | Yeast Culture Kit Company |
| 39 | ACY39 | Raspberry - stem | Isolated from Iowa, US | This study |
| 40 | ACY40 | Raspberry - soil | Isolated from Iowa, US | This study |
| 41 | ACY41 | Cottonwood - bark #1 | Isolated from Iowa, US | This study |
| 42 | ACY42 | Cottonwood - bark #2 | Isolated from Iowa, US | This study |
| 43 | ACY43 | Cottonwood - soil | Isolated from Iowa, US | This study |

## Slide 3
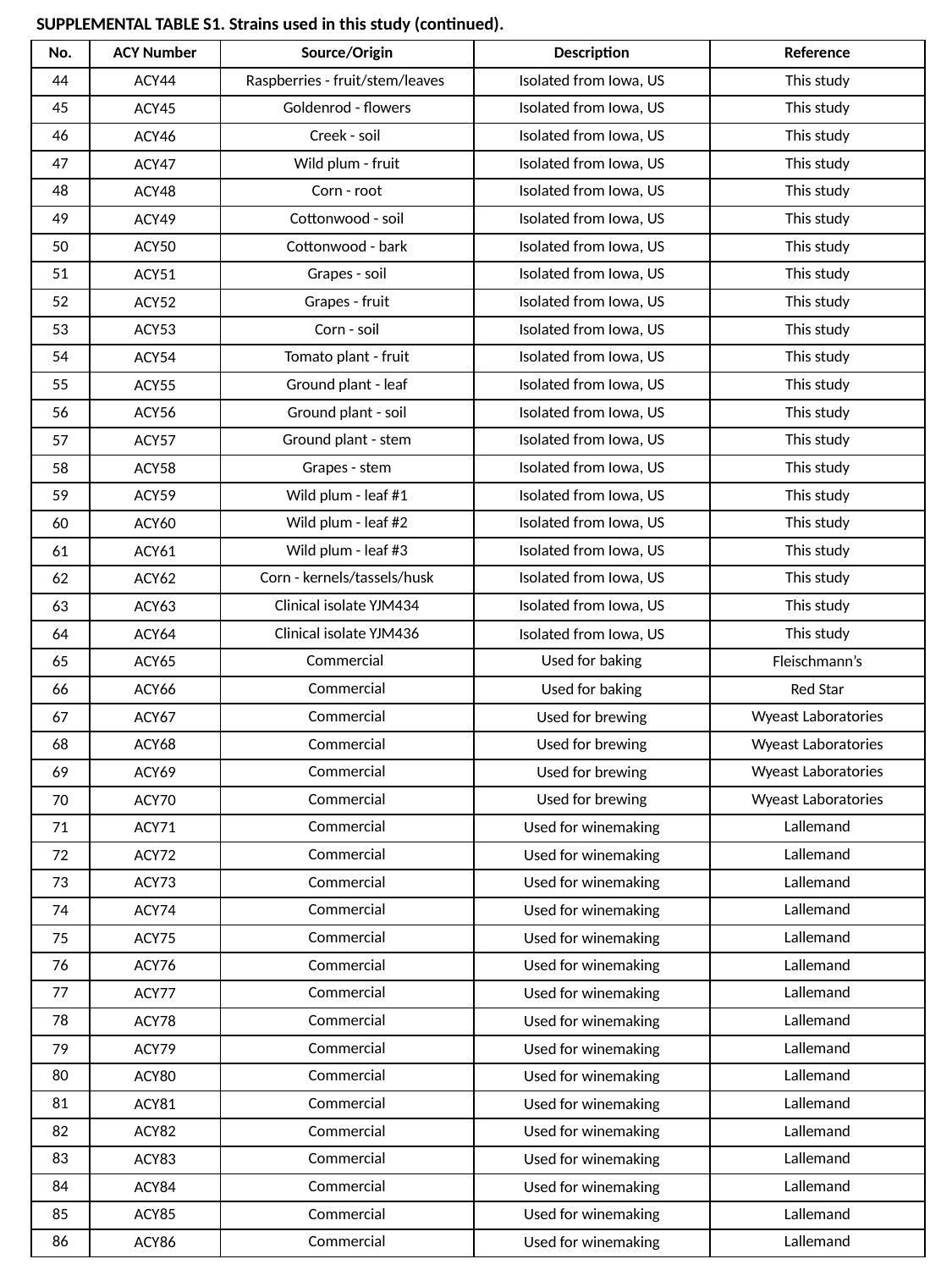

SUPPLEMENTAL TABLE S1. Strains used in this study (continued).
| No. | ACY Number | Source/Origin | Description | Reference |
| --- | --- | --- | --- | --- |
| 44 | ACY44 | Raspberries - fruit/stem/leaves | Isolated from Iowa, US | This study |
| 45 | ACY45 | Goldenrod - flowers | Isolated from Iowa, US | This study |
| 46 | ACY46 | Creek - soil | Isolated from Iowa, US | This study |
| 47 | ACY47 | Wild plum - fruit | Isolated from Iowa, US | This study |
| 48 | ACY48 | Corn - root | Isolated from Iowa, US | This study |
| 49 | ACY49 | Cottonwood - soil | Isolated from Iowa, US | This study |
| 50 | ACY50 | Cottonwood - bark | Isolated from Iowa, US | This study |
| 51 | ACY51 | Grapes - soil | Isolated from Iowa, US | This study |
| 52 | ACY52 | Grapes - fruit | Isolated from Iowa, US | This study |
| 53 | ACY53 | Corn - soil | Isolated from Iowa, US | This study |
| 54 | ACY54 | Tomato plant - fruit | Isolated from Iowa, US | This study |
| 55 | ACY55 | Ground plant - leaf | Isolated from Iowa, US | This study |
| 56 | ACY56 | Ground plant - soil | Isolated from Iowa, US | This study |
| 57 | ACY57 | Ground plant - stem | Isolated from Iowa, US | This study |
| 58 | ACY58 | Grapes - stem | Isolated from Iowa, US | This study |
| 59 | ACY59 | Wild plum - leaf #1 | Isolated from Iowa, US | This study |
| 60 | ACY60 | Wild plum - leaf #2 | Isolated from Iowa, US | This study |
| 61 | ACY61 | Wild plum - leaf #3 | Isolated from Iowa, US | This study |
| 62 | ACY62 | Corn - kernels/tassels/husk | Isolated from Iowa, US | This study |
| 63 | ACY63 | Clinical isolate YJM434 | Isolated from Iowa, US | This study |
| 64 | ACY64 | Clinical isolate YJM436 | Isolated from Iowa, US | This study |
| 65 | ACY65 | Commercial | Used for baking | Fleischmann’s |
| 66 | ACY66 | Commercial | Used for baking | Red Star |
| 67 | ACY67 | Commercial | Used for brewing | Wyeast Laboratories |
| 68 | ACY68 | Commercial | Used for brewing | Wyeast Laboratories |
| 69 | ACY69 | Commercial | Used for brewing | Wyeast Laboratories |
| 70 | ACY70 | Commercial | Used for brewing | Wyeast Laboratories |
| 71 | ACY71 | Commercial | Used for winemaking | Lallemand |
| 72 | ACY72 | Commercial | Used for winemaking | Lallemand |
| 73 | ACY73 | Commercial | Used for winemaking | Lallemand |
| 74 | ACY74 | Commercial | Used for winemaking | Lallemand |
| 75 | ACY75 | Commercial | Used for winemaking | Lallemand |
| 76 | ACY76 | Commercial | Used for winemaking | Lallemand |
| 77 | ACY77 | Commercial | Used for winemaking | Lallemand |
| 78 | ACY78 | Commercial | Used for winemaking | Lallemand |
| 79 | ACY79 | Commercial | Used for winemaking | Lallemand |
| 80 | ACY80 | Commercial | Used for winemaking | Lallemand |
| 81 | ACY81 | Commercial | Used for winemaking | Lallemand |
| 82 | ACY82 | Commercial | Used for winemaking | Lallemand |
| 83 | ACY83 | Commercial | Used for winemaking | Lallemand |
| 84 | ACY84 | Commercial | Used for winemaking | Lallemand |
| 85 | ACY85 | Commercial | Used for winemaking | Lallemand |
| 86 | ACY86 | Commercial | Used for winemaking | Lallemand |

## Slide 4
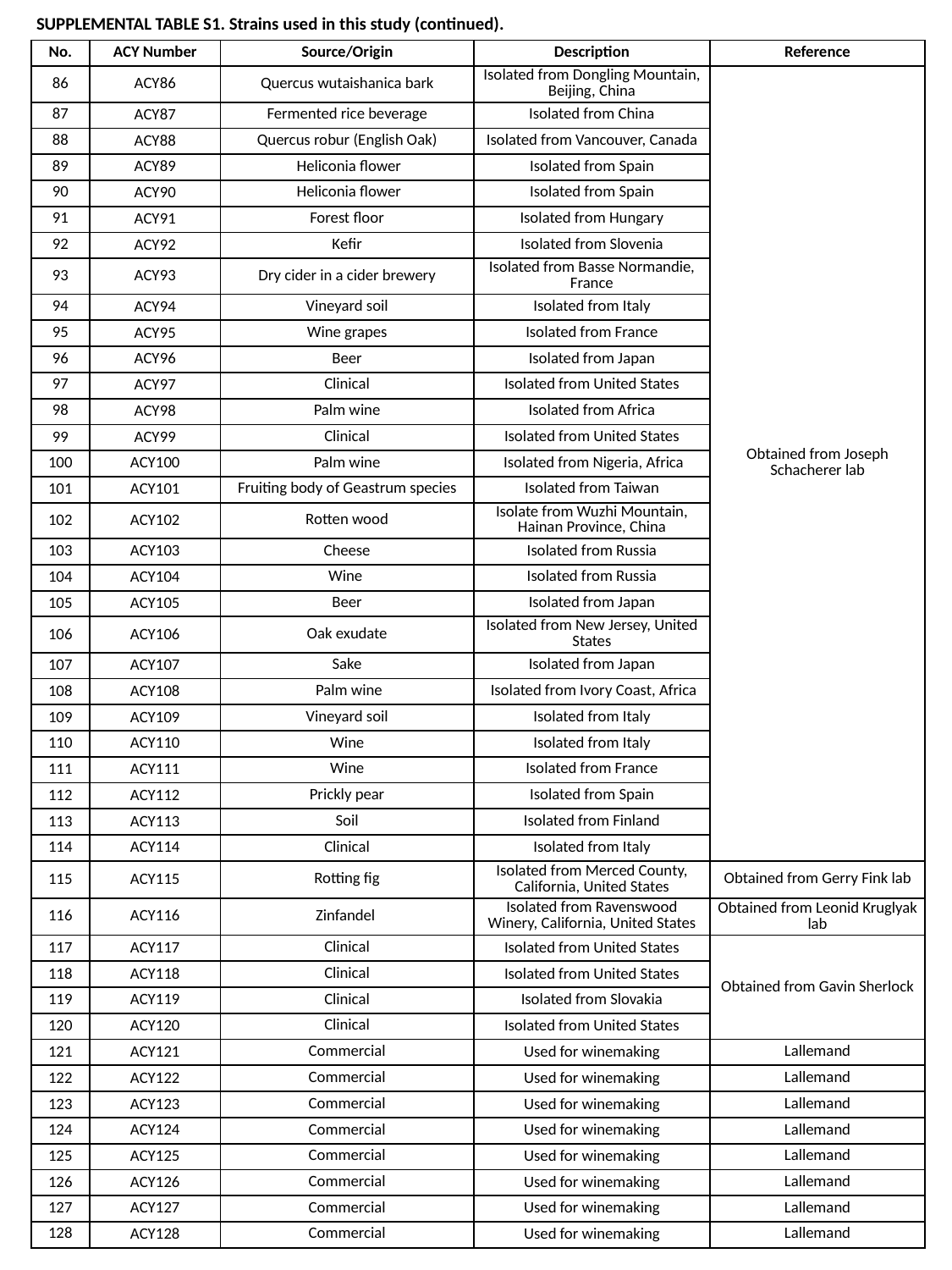

SUPPLEMENTAL TABLE S1. Strains used in this study (continued).
| No. | ACY Number | Source/Origin | Description | Reference |
| --- | --- | --- | --- | --- |
| 86 | ACY86 | Quercus wutaishanica bark | Isolated from Dongling Mountain, Beijing, China | Obtained from Joseph Schacherer lab |
| 87 | ACY87 | Fermented rice beverage | Isolated from China | |
| 88 | ACY88 | Quercus robur (English Oak) | Isolated from Vancouver, Canada | |
| 89 | ACY89 | Heliconia flower | Isolated from Spain | |
| 90 | ACY90 | Heliconia flower | Isolated from Spain | |
| 91 | ACY91 | Forest floor | Isolated from Hungary | |
| 92 | ACY92 | Kefir | Isolated from Slovenia | |
| 93 | ACY93 | Dry cider in a cider brewery | Isolated from Basse Normandie, France | |
| 94 | ACY94 | Vineyard soil | Isolated from Italy | |
| 95 | ACY95 | Wine grapes | Isolated from France | |
| 96 | ACY96 | Beer | Isolated from Japan | |
| 97 | ACY97 | Clinical | Isolated from United States | |
| 98 | ACY98 | Palm wine | Isolated from Africa | |
| 99 | ACY99 | Clinical | Isolated from United States | |
| 100 | ACY100 | Palm wine | Isolated from Nigeria, Africa | |
| 101 | ACY101 | Fruiting body of Geastrum species | Isolated from Taiwan | |
| 102 | ACY102 | Rotten wood | Isolate from Wuzhi Mountain, Hainan Province, China | |
| 103 | ACY103 | Cheese | Isolated from Russia | |
| 104 | ACY104 | Wine | Isolated from Russia | |
| 105 | ACY105 | Beer | Isolated from Japan | |
| 106 | ACY106 | Oak exudate | Isolated from New Jersey, United States | |
| 107 | ACY107 | Sake | Isolated from Japan | |
| 108 | ACY108 | Palm wine | Isolated from Ivory Coast, Africa | |
| 109 | ACY109 | Vineyard soil | Isolated from Italy | |
| 110 | ACY110 | Wine | Isolated from Italy | |
| 111 | ACY111 | Wine | Isolated from France | |
| 112 | ACY112 | Prickly pear | Isolated from Spain | |
| 113 | ACY113 | Soil | Isolated from Finland | |
| 114 | ACY114 | Clinical | Isolated from Italy | |
| 115 | ACY115 | Rotting fig | Isolated from Merced County, California, United States | Obtained from Gerry Fink lab |
| 116 | ACY116 | Zinfandel | Isolated from Ravenswood Winery, California, United States | Obtained from Leonid Kruglyak lab |
| 117 | ACY117 | Clinical | Isolated from United States | Obtained from Gavin Sherlock |
| 118 | ACY118 | Clinical | Isolated from United States | |
| 119 | ACY119 | Clinical | Isolated from Slovakia | |
| 120 | ACY120 | Clinical | Isolated from United States | |
| 121 | ACY121 | Commercial | Used for winemaking | Lallemand |
| 122 | ACY122 | Commercial | Used for winemaking | Lallemand |
| 123 | ACY123 | Commercial | Used for winemaking | Lallemand |
| 124 | ACY124 | Commercial | Used for winemaking | Lallemand |
| 125 | ACY125 | Commercial | Used for winemaking | Lallemand |
| 126 | ACY126 | Commercial | Used for winemaking | Lallemand |
| 127 | ACY127 | Commercial | Used for winemaking | Lallemand |
| 128 | ACY128 | Commercial | Used for winemaking | Lallemand |

## Slide 5
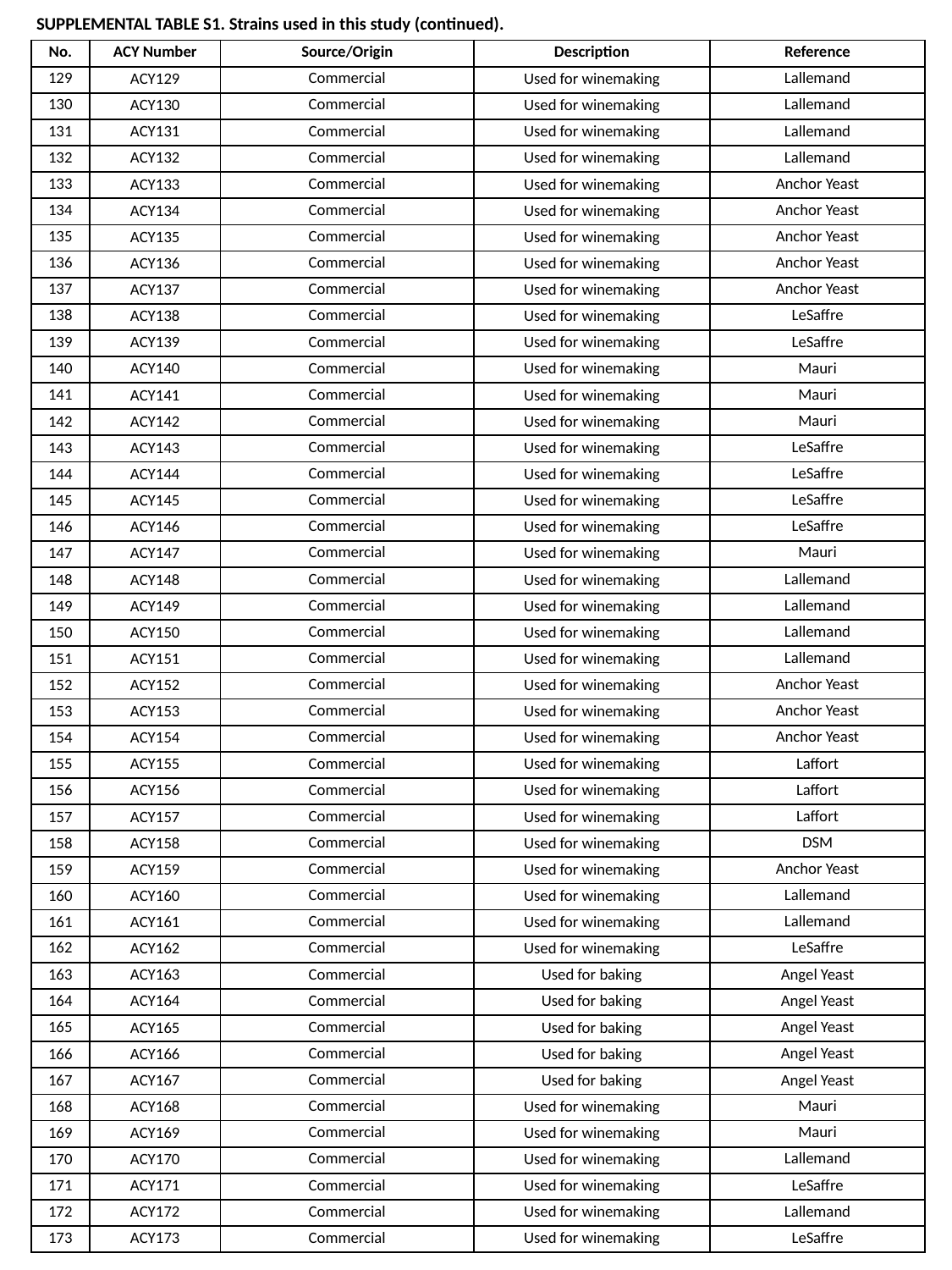

SUPPLEMENTAL TABLE S1. Strains used in this study (continued).
| No. | ACY Number | Source/Origin | Description | Reference |
| --- | --- | --- | --- | --- |
| 129 | ACY129 | Commercial | Used for winemaking | Lallemand |
| 130 | ACY130 | Commercial | Used for winemaking | Lallemand |
| 131 | ACY131 | Commercial | Used for winemaking | Lallemand |
| 132 | ACY132 | Commercial | Used for winemaking | Lallemand |
| 133 | ACY133 | Commercial | Used for winemaking | Anchor Yeast |
| 134 | ACY134 | Commercial | Used for winemaking | Anchor Yeast |
| 135 | ACY135 | Commercial | Used for winemaking | Anchor Yeast |
| 136 | ACY136 | Commercial | Used for winemaking | Anchor Yeast |
| 137 | ACY137 | Commercial | Used for winemaking | Anchor Yeast |
| 138 | ACY138 | Commercial | Used for winemaking | LeSaffre |
| 139 | ACY139 | Commercial | Used for winemaking | LeSaffre |
| 140 | ACY140 | Commercial | Used for winemaking | Mauri |
| 141 | ACY141 | Commercial | Used for winemaking | Mauri |
| 142 | ACY142 | Commercial | Used for winemaking | Mauri |
| 143 | ACY143 | Commercial | Used for winemaking | LeSaffre |
| 144 | ACY144 | Commercial | Used for winemaking | LeSaffre |
| 145 | ACY145 | Commercial | Used for winemaking | LeSaffre |
| 146 | ACY146 | Commercial | Used for winemaking | LeSaffre |
| 147 | ACY147 | Commercial | Used for winemaking | Mauri |
| 148 | ACY148 | Commercial | Used for winemaking | Lallemand |
| 149 | ACY149 | Commercial | Used for winemaking | Lallemand |
| 150 | ACY150 | Commercial | Used for winemaking | Lallemand |
| 151 | ACY151 | Commercial | Used for winemaking | Lallemand |
| 152 | ACY152 | Commercial | Used for winemaking | Anchor Yeast |
| 153 | ACY153 | Commercial | Used for winemaking | Anchor Yeast |
| 154 | ACY154 | Commercial | Used for winemaking | Anchor Yeast |
| 155 | ACY155 | Commercial | Used for winemaking | Laffort |
| 156 | ACY156 | Commercial | Used for winemaking | Laffort |
| 157 | ACY157 | Commercial | Used for winemaking | Laffort |
| 158 | ACY158 | Commercial | Used for winemaking | DSM |
| 159 | ACY159 | Commercial | Used for winemaking | Anchor Yeast |
| 160 | ACY160 | Commercial | Used for winemaking | Lallemand |
| 161 | ACY161 | Commercial | Used for winemaking | Lallemand |
| 162 | ACY162 | Commercial | Used for winemaking | LeSaffre |
| 163 | ACY163 | Commercial | Used for baking | Angel Yeast |
| 164 | ACY164 | Commercial | Used for baking | Angel Yeast |
| 165 | ACY165 | Commercial | Used for baking | Angel Yeast |
| 166 | ACY166 | Commercial | Used for baking | Angel Yeast |
| 167 | ACY167 | Commercial | Used for baking | Angel Yeast |
| 168 | ACY168 | Commercial | Used for winemaking | Mauri |
| 169 | ACY169 | Commercial | Used for winemaking | Mauri |
| 170 | ACY170 | Commercial | Used for winemaking | Lallemand |
| 171 | ACY171 | Commercial | Used for winemaking | LeSaffre |
| 172 | ACY172 | Commercial | Used for winemaking | Lallemand |
| 173 | ACY173 | Commercial | Used for winemaking | LeSaffre |

## Slide 6
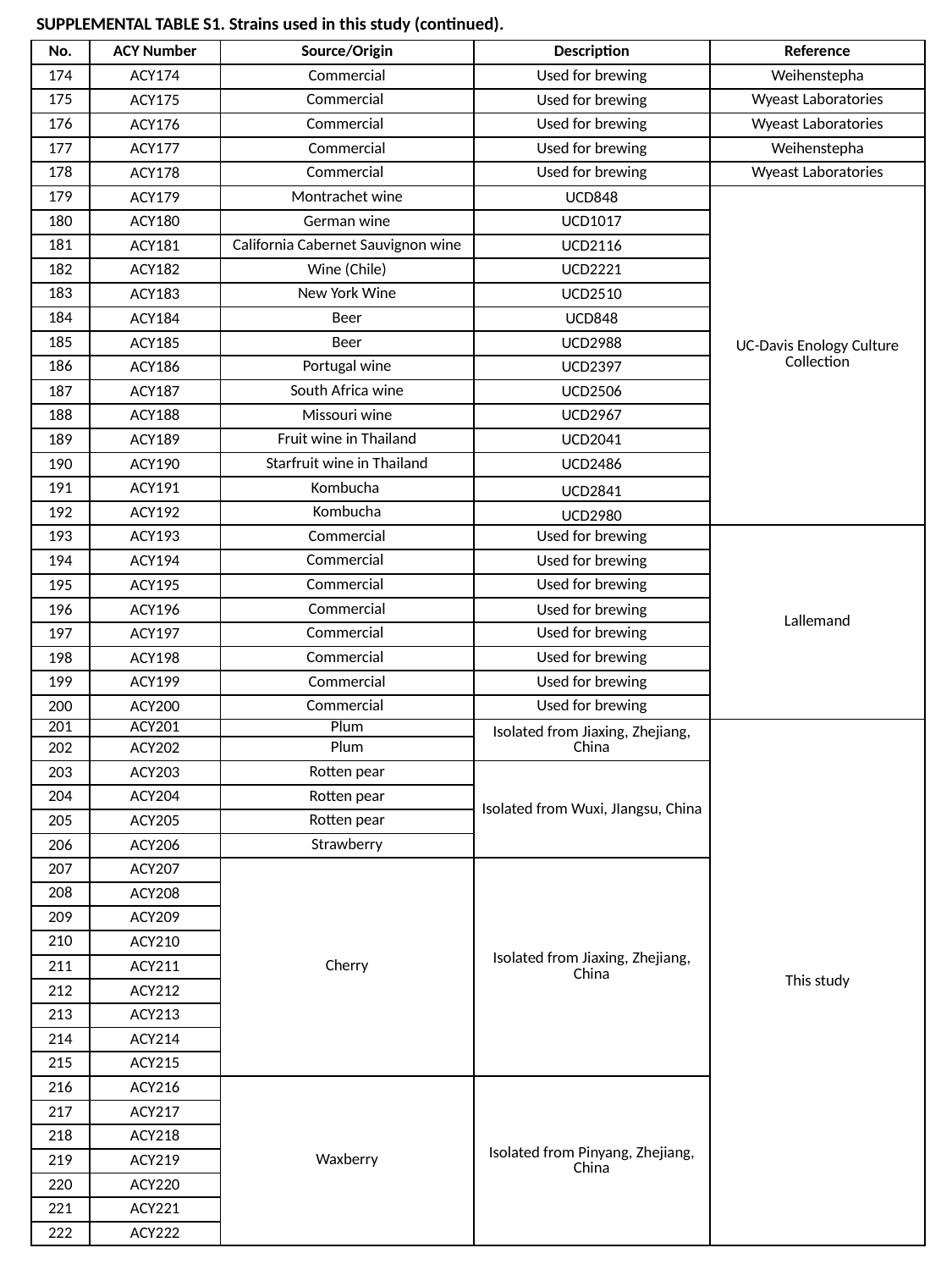

SUPPLEMENTAL TABLE S1. Strains used in this study (continued).
| No. | ACY Number | Source/Origin | Description | Reference |
| --- | --- | --- | --- | --- |
| 174 | ACY174 | Commercial | Used for brewing | Weihenstepha |
| 175 | ACY175 | Commercial | Used for brewing | Wyeast Laboratories |
| 176 | ACY176 | Commercial | Used for brewing | Wyeast Laboratories |
| 177 | ACY177 | Commercial | Used for brewing | Weihenstepha |
| 178 | ACY178 | Commercial | Used for brewing | Wyeast Laboratories |
| 179 | ACY179 | Montrachet wine | UCD848 | UC-Davis Enology Culture Collection |
| 180 | ACY180 | German wine | UCD1017 | |
| 181 | ACY181 | California Cabernet Sauvignon wine | UCD2116 | |
| 182 | ACY182 | Wine (Chile) | UCD2221 | |
| 183 | ACY183 | New York Wine | UCD2510 | |
| 184 | ACY184 | Beer | UCD848 | |
| 185 | ACY185 | Beer | UCD2988 | |
| 186 | ACY186 | Portugal wine | UCD2397 | |
| 187 | ACY187 | South Africa wine | UCD2506 | |
| 188 | ACY188 | Missouri wine | UCD2967 | |
| 189 | ACY189 | Fruit wine in Thailand | UCD2041 | |
| 190 | ACY190 | Starfruit wine in Thailand | UCD2486 | |
| 191 | ACY191 | Kombucha | UCD2841 | |
| 192 | ACY192 | Kombucha | UCD2980 | |
| 193 | ACY193 | Commercial | Used for brewing | Lallemand |
| 194 | ACY194 | Commercial | Used for brewing | |
| 195 | ACY195 | Commercial | Used for brewing | |
| 196 | ACY196 | Commercial | Used for brewing | |
| 197 | ACY197 | Commercial | Used for brewing | |
| 198 | ACY198 | Commercial | Used for brewing | |
| 199 | ACY199 | Commercial | Used for brewing | |
| 200 | ACY200 | Commercial | Used for brewing | |
| 201 | ACY201 | Plum | Isolated from Jiaxing, Zhejiang, China | This study |
| 202 | ACY202 | Plum | | |
| 203 | ACY203 | Rotten pear | Isolated from Wuxi, JIangsu, China | |
| 204 | ACY204 | Rotten pear | | |
| 205 | ACY205 | Rotten pear | | |
| 206 | ACY206 | Strawberry | | |
| 207 | ACY207 | Cherry | Isolated from Jiaxing, Zhejiang, China | |
| 208 | ACY208 | | | |
| 209 | ACY209 | | | |
| 210 | ACY210 | | | |
| 211 | ACY211 | | | |
| 212 | ACY212 | | | |
| 213 | ACY213 | | | |
| 214 | ACY214 | | | |
| 215 | ACY215 | | | |
| 216 | ACY216 | Waxberry | Isolated from Pinyang, Zhejiang, China | |
| 217 | ACY217 | | | |
| 218 | ACY218 | | | |
| 219 | ACY219 | | | |
| 220 | ACY220 | | | |
| 221 | ACY221 | | | |
| 222 | ACY222 | | | |

## Slide 7
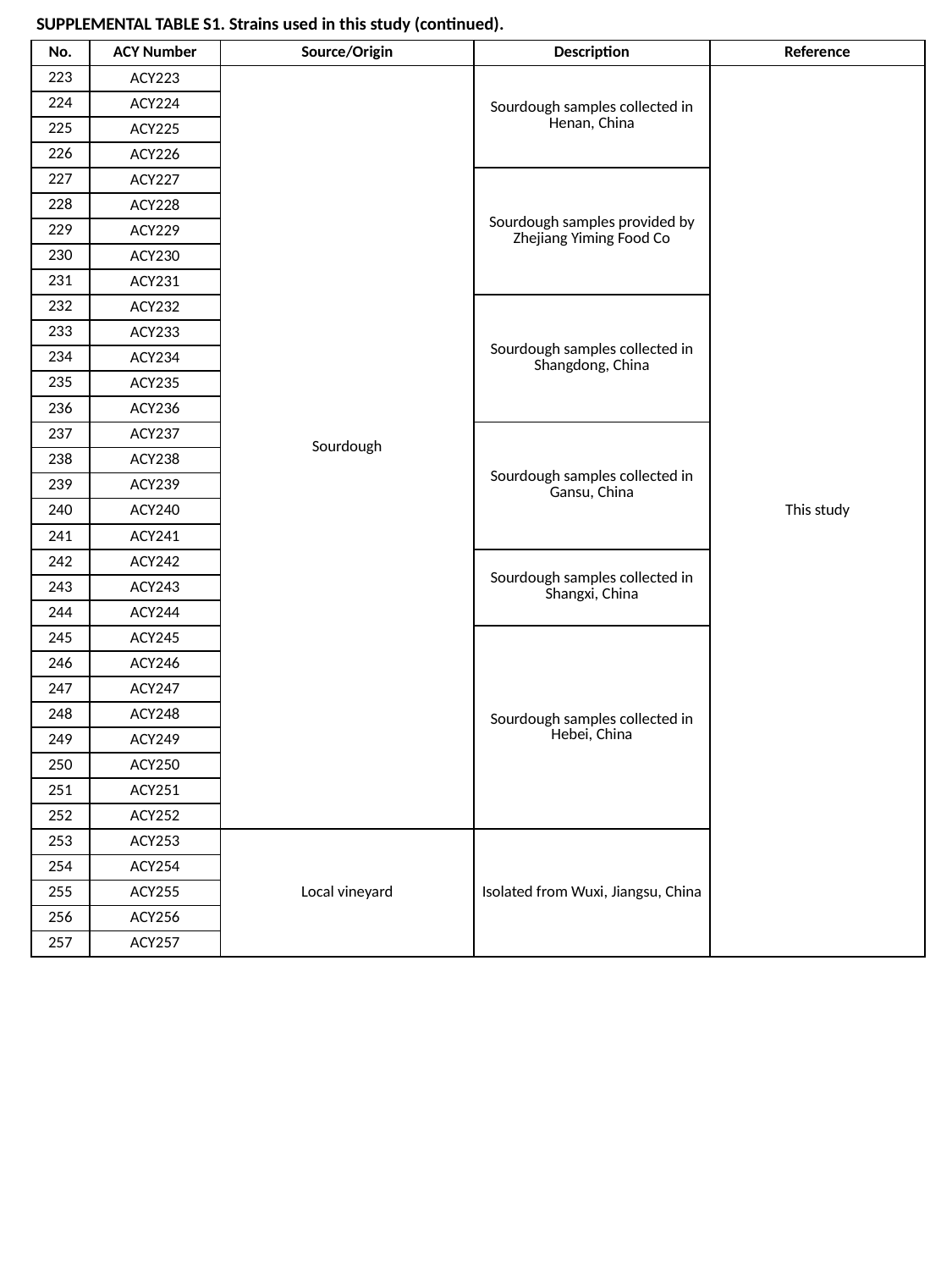

SUPPLEMENTAL TABLE S1. Strains used in this study (continued).
| No. | ACY Number | Source/Origin | Description | Reference |
| --- | --- | --- | --- | --- |
| 223 | ACY223 | Sourdough | Sourdough samples collected in Henan, China | This study |
| 224 | ACY224 | | | |
| 225 | ACY225 | | | |
| 226 | ACY226 | | | |
| 227 | ACY227 | | Sourdough samples provided by Zhejiang Yiming Food Co | |
| 228 | ACY228 | | | |
| 229 | ACY229 | | | |
| 230 | ACY230 | | | |
| 231 | ACY231 | | | |
| 232 | ACY232 | | Sourdough samples collected in Shangdong, China | |
| 233 | ACY233 | | | |
| 234 | ACY234 | | | |
| 235 | ACY235 | | | |
| 236 | ACY236 | | | |
| 237 | ACY237 | | Sourdough samples collected in Gansu, China | |
| 238 | ACY238 | | | |
| 239 | ACY239 | | | |
| 240 | ACY240 | | | |
| 241 | ACY241 | | | |
| 242 | ACY242 | | Sourdough samples collected in Shangxi, China | |
| 243 | ACY243 | | | |
| 244 | ACY244 | | | |
| 245 | ACY245 | | Sourdough samples collected in Hebei, China | |
| 246 | ACY246 | | | |
| 247 | ACY247 | | | |
| 248 | ACY248 | | | |
| 249 | ACY249 | | | |
| 250 | ACY250 | | | |
| 251 | ACY251 | | | |
| 252 | ACY252 | | | |
| 253 | ACY253 | Local vineyard | Isolated from Wuxi, Jiangsu, China | |
| 254 | ACY254 | | | |
| 255 | ACY255 | | | |
| 256 | ACY256 | | | |
| 257 | ACY257 | | | |

## Slide 8
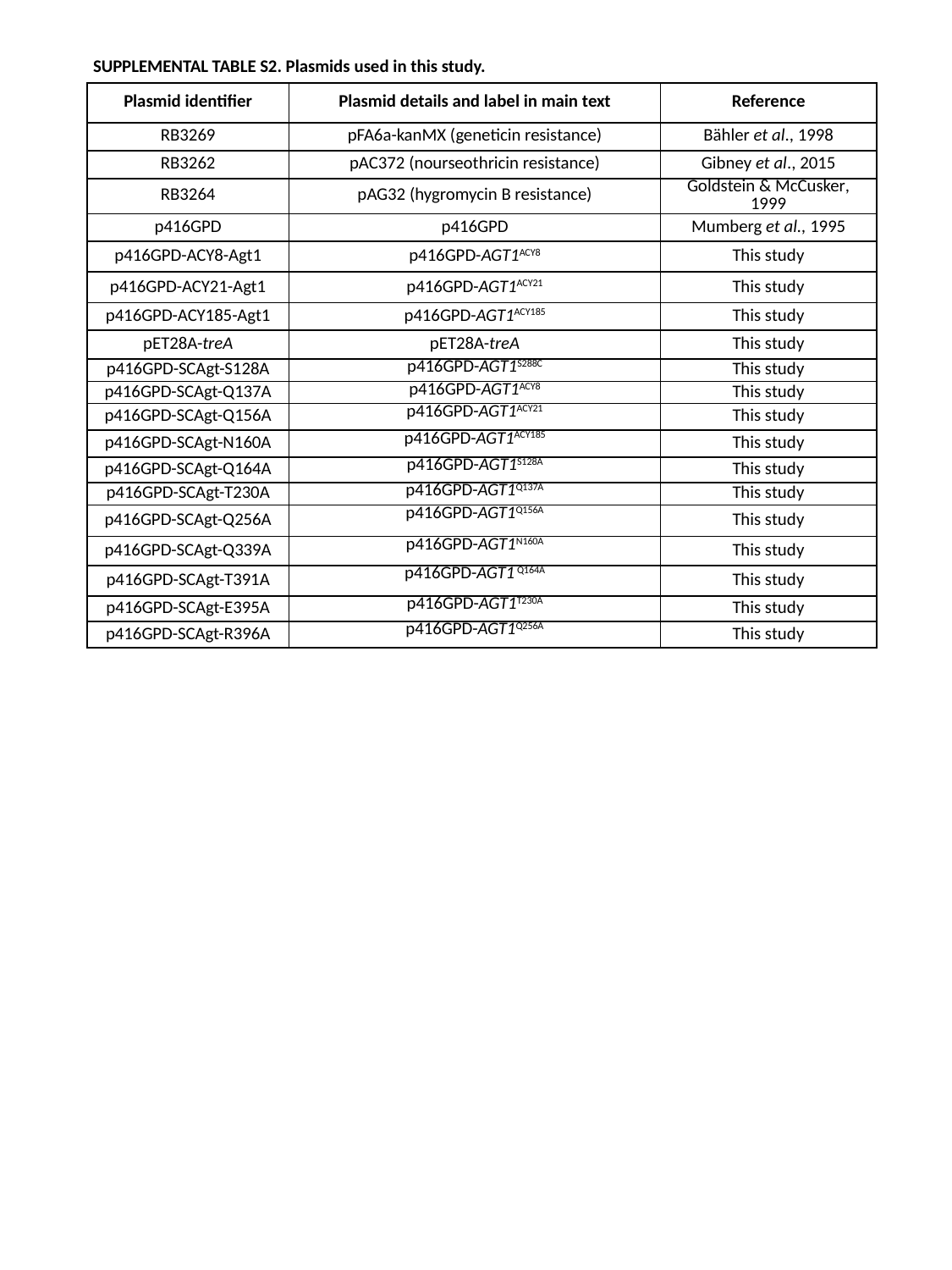

SUPPLEMENTAL TABLE S2. Plasmids used in this study.
| Plasmid identifier | Plasmid details and label in main text | Reference |
| --- | --- | --- |
| RB3269 | pFA6a-kanMX (geneticin resistance) | Bähler et al., 1998 |
| RB3262 | pAC372 (nourseothricin resistance) | Gibney et al., 2015 |
| RB3264 | pAG32 (hygromycin B resistance) | Goldstein & McCusker, 1999 |
| p416GPD | p416GPD | Mumberg et al., 1995 |
| p416GPD-ACY8-Agt1 | p416GPD-AGT1ACY8 | This study |
| p416GPD-ACY21-Agt1 | p416GPD-AGT1ACY21 | This study |
| p416GPD-ACY185-Agt1 | p416GPD-AGT1ACY185 | This study |
| pET28A-treA | pET28A-treA | This study |
| p416GPD-SCAgt-S128A | p416GPD-AGT1S288C | This study |
| p416GPD-SCAgt-Q137A | p416GPD-AGT1ACY8 | This study |
| p416GPD-SCAgt-Q156A | p416GPD-AGT1ACY21 | This study |
| p416GPD-SCAgt-N160A | p416GPD-AGT1ACY185 | This study |
| p416GPD-SCAgt-Q164A | p416GPD-AGT1S128A | This study |
| p416GPD-SCAgt-T230A | p416GPD-AGT1Q137A | This study |
| p416GPD-SCAgt-Q256A | p416GPD-AGT1Q156A | This study |
| p416GPD-SCAgt-Q339A | p416GPD-AGT1N160A | This study |
| p416GPD-SCAgt-T391A | p416GPD-AGT1 Q164A | This study |
| p416GPD-SCAgt-E395A | p416GPD-AGT1T230A | This study |
| p416GPD-SCAgt-R396A | p416GPD-AGT1Q256A | This study |

## Slide 9
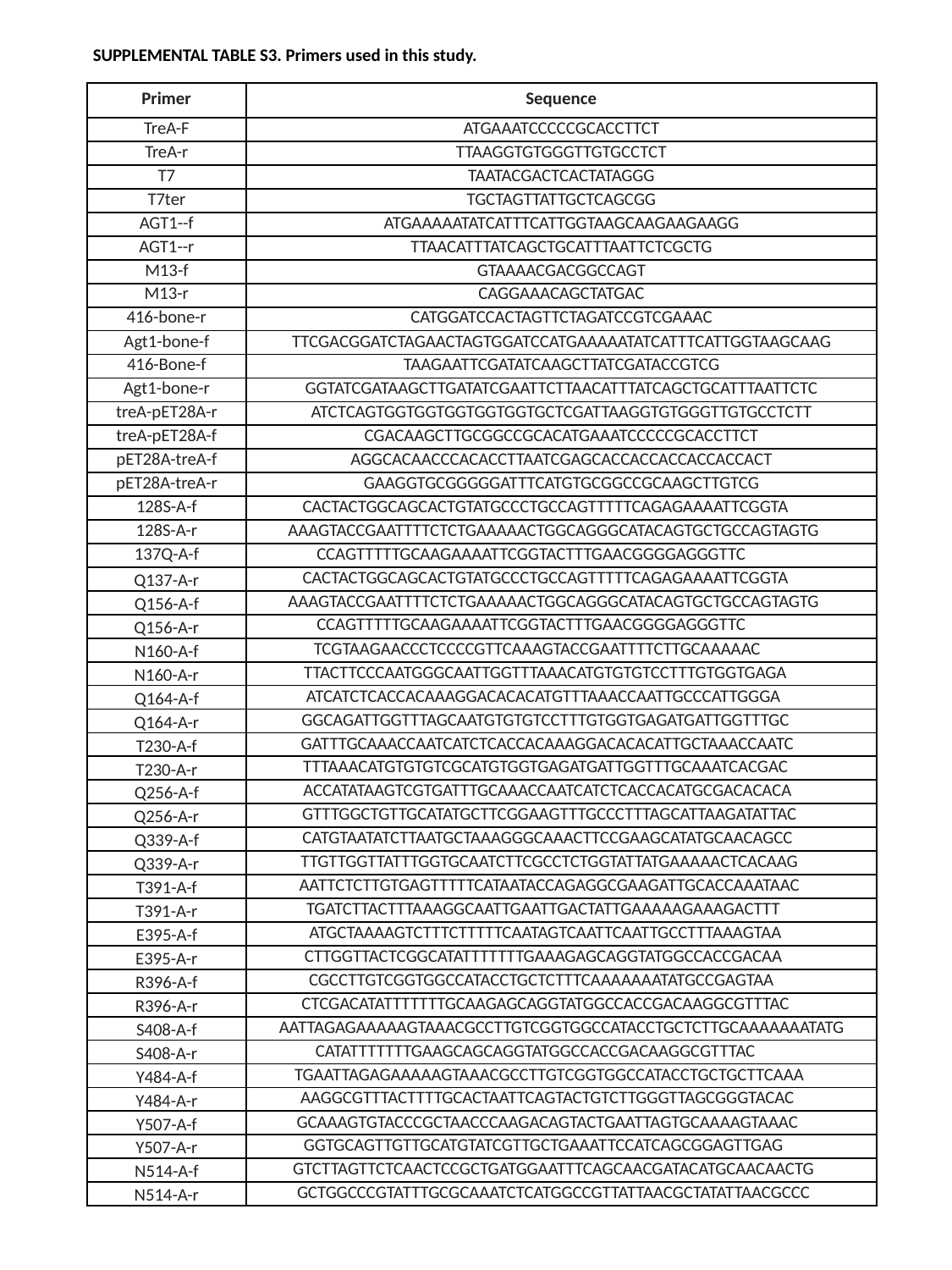

SUPPLEMENTAL TABLE S3. Primers used in this study.
| Primer | Sequence |
| --- | --- |
| TreA-F | ATGAAATCCCCCGCACCTTCT |
| TreA-r | TTAAGGTGTGGGTTGTGCCTCT |
| T7 | TAATACGACTCACTATAGGG |
| T7ter | TGCTAGTTATTGCTCAGCGG |
| AGT1--f | ATGAAAAATATCATTTCATTGGTAAGCAAGAAGAAGG |
| AGT1--r | TTAACATTTATCAGCTGCATTTAATTCTCGCTG |
| M13-f | GTAAAACGACGGCCAGT |
| M13-r | CAGGAAACAGCTATGAC |
| 416-bone-r | CATGGATCCACTAGTTCTAGATCCGTCGAAAC |
| Agt1-bone-f | TTCGACGGATCTAGAACTAGTGGATCCATGAAAAATATCATTTCATTGGTAAGCAAG |
| 416-Bone-f | TAAGAATTCGATATCAAGCTTATCGATACCGTCG |
| Agt1-bone-r | GGTATCGATAAGCTTGATATCGAATTCTTAACATTTATCAGCTGCATTTAATTCTC |
| treA-pET28A-r | ATCTCAGTGGTGGTGGTGGTGGTGCTCGATTAAGGTGTGGGTTGTGCCTCTT |
| treA-pET28A-f | CGACAAGCTTGCGGCCGCACATGAAATCCCCCGCACCTTCT |
| pET28A-treA-f | AGGCACAACCCACACCTTAATCGAGCACCACCACCACCACCACT |
| pET28A-treA-r | GAAGGTGCGGGGGATTTCATGTGCGGCCGCAAGCTTGTCG |
| 128S-A-f | CACTACTGGCAGCACTGTATGCCCTGCCAGTTTTTCAGAGAAAATTCGGTA |
| 128S-A-r | AAAGTACCGAATTTTCTCTGAAAAACTGGCAGGGCATACAGTGCTGCCAGTAGTG |
| 137Q-A-f | CCAGTTTTTGCAAGAAAATTCGGTACTTTGAACGGGGAGGGTTC |
| Q137-A-r | CACTACTGGCAGCACTGTATGCCCTGCCAGTTTTTCAGAGAAAATTCGGTA |
| Q156-A-f | AAAGTACCGAATTTTCTCTGAAAAACTGGCAGGGCATACAGTGCTGCCAGTAGTG |
| Q156-A-r | CCAGTTTTTGCAAGAAAATTCGGTACTTTGAACGGGGAGGGTTC |
| N160-A-f | TCGTAAGAACCCTCCCCGTTCAAAGTACCGAATTTTCTTGCAAAAAC |
| N160-A-r | TTACTTCCCAATGGGCAATTGGTTTAAACATGTGTGTCCTTTGTGGTGAGA |
| Q164-A-f | ATCATCTCACCACAAAGGACACACATGTTTAAACCAATTGCCCATTGGGA |
| Q164-A-r | GGCAGATTGGTTTAGCAATGTGTGTCCTTTGTGGTGAGATGATTGGTTTGC |
| T230-A-f | GATTTGCAAACCAATCATCTCACCACAAAGGACACACATTGCTAAACCAATC |
| T230-A-r | TTTAAACATGTGTGTCGCATGTGGTGAGATGATTGGTTTGCAAATCACGAC |
| Q256-A-f | ACCATATAAGTCGTGATTTGCAAACCAATCATCTCACCACATGCGACACACA |
| Q256-A-r | GTTTGGCTGTTGCATATGCTTCGGAAGTTTGCCCTTTAGCATTAAGATATTAC |
| Q339-A-f | CATGTAATATCTTAATGCTAAAGGGCAAACTTCCGAAGCATATGCAACAGCC |
| Q339-A-r | TTGTTGGTTATTTGGTGCAATCTTCGCCTCTGGTATTATGAAAAACTCACAAG |
| T391-A-f | AATTCTCTTGTGAGTTTTTCATAATACCAGAGGCGAAGATTGCACCAAATAAC |
| T391-A-r | TGATCTTACTTTAAAGGCAATTGAATTGACTATTGAAAAAGAAAGACTTT |
| E395-A-f | ATGCTAAAAGTCTTTCTTTTTCAATAGTCAATTCAATTGCCTTTAAAGTAA |
| E395-A-r | CTTGGTTACTCGGCATATTTTTTTGAAAGAGCAGGTATGGCCACCGACAA |
| R396-A-f | CGCCTTGTCGGTGGCCATACCTGCTCTTTCAAAAAAATATGCCGAGTAA |
| R396-A-r | CTCGACATATTTTTTTGCAAGAGCAGGTATGGCCACCGACAAGGCGTTTAC |
| S408-A-f | AATTAGAGAAAAAGTAAACGCCTTGTCGGTGGCCATACCTGCTCTTGCAAAAAAATATG |
| S408-A-r | CATATTTTTTTGAAGCAGCAGGTATGGCCACCGACAAGGCGTTTAC |
| Y484-A-f | TGAATTAGAGAAAAAGTAAACGCCTTGTCGGTGGCCATACCTGCTGCTTCAAA |
| Y484-A-r | AAGGCGTTTACTTTTGCACTAATTCAGTACTGTCTTGGGTTAGCGGGTACAC |
| Y507-A-f | GCAAAGTGTACCCGCTAACCCAAGACAGTACTGAATTAGTGCAAAAGTAAAC |
| Y507-A-r | GGTGCAGTTGTTGCATGTATCGTTGCTGAAATTCCATCAGCGGAGTTGAG |
| N514-A-f | GTCTTAGTTCTCAACTCCGCTGATGGAATTTCAGCAACGATACATGCAACAACTG |
| N514-A-r | GCTGGCCCGTATTTGCGCAAATCTCATGGCCGTTATTAACGCTATATTAACGCCC |

## Slide 10
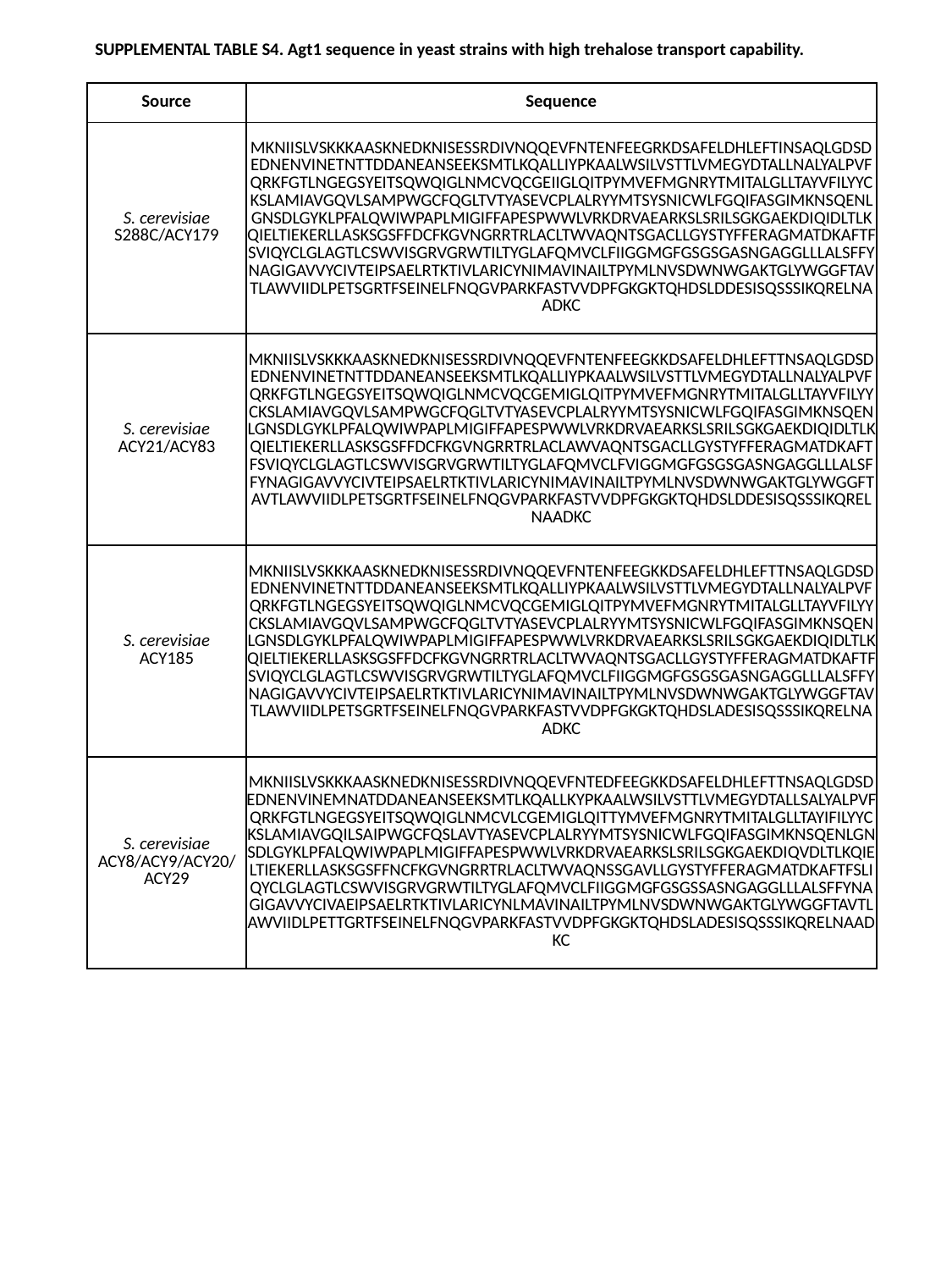

SUPPLEMENTAL TABLE S4. Agt1 sequence in yeast strains with high trehalose transport capability.
| Source | Sequence |
| --- | --- |
| S. cerevisiae S288C/ACY179 | MKNIISLVSKKKAASKNEDKNISESSRDIVNQQEVFNTENFEEGRKDSAFELDHLEFTINSAQLGDSDEDNENVINETNTTDDANEANSEEKSMTLKQALLIYPKAALWSILVSTTLVMEGYDTALLNALYALPVFQRKFGTLNGEGSYEITSQWQIGLNMCVQCGEIIGLQITPYMVEFMGNRYTMITALGLLTAYVFILYYCKSLAMIAVGQVLSAMPWGCFQGLTVTYASEVCPLALRYYMTSYSNICWLFGQIFASGIMKNSQENLGNSDLGYKLPFALQWIWPAPLMIGIFFAPESPWWLVRKDRVAEARKSLSRILSGKGAEKDIQIDLTLKQIELTIEKERLLASKSGSFFDCFKGVNGRRTRLACLTWVAQNTSGACLLGYSTYFFERAGMATDKAFTFSVIQYCLGLAGTLCSWVISGRVGRWTILTYGLAFQMVCLFIIGGMGFGSGSGASNGAGGLLLALSFFYNAGIGAVVYCIVTEIPSAELRTKTIVLARICYNIMAVINAILTPYMLNVSDWNWGAKTGLYWGGFTAVTLAWVIIDLPETSGRTFSEINELFNQGVPARKFASTVVDPFGKGKTQHDSLDDESISQSSSIKQRELNAADKC |
| S. cerevisiae ACY21/ACY83 | MKNIISLVSKKKAASKNEDKNISESSRDIVNQQEVFNTENFEEGKKDSAFELDHLEFTTNSAQLGDSDEDNENVINETNTTDDANEANSEEKSMTLKQALLIYPKAALWSILVSTTLVMEGYDTALLNALYALPVFQRKFGTLNGEGSYEITSQWQIGLNMCVQCGEMIGLQITPYMVEFMGNRYTMITALGLLTAYVFILYYCKSLAMIAVGQVLSAMPWGCFQGLTVTYASEVCPLALRYYMTSYSNICWLFGQIFASGIMKNSQENLGNSDLGYKLPFALQWIWPAPLMIGIFFAPESPWWLVRKDRVAEARKSLSRILSGKGAEKDIQIDLTLKQIELTIEKERLLASKSGSFFDCFKGVNGRRTRLACLAWVAQNTSGACLLGYSTYFFERAGMATDKAFTFSVIQYCLGLAGTLCSWVISGRVGRWTILTYGLAFQMVCLFVIGGMGFGSGSGASNGAGGLLLALSFFYNAGIGAVVYCIVTEIPSAELRTKTIVLARICYNIMAVINAILTPYMLNVSDWNWGAKTGLYWGGFTAVTLAWVIIDLPETSGRTFSEINELFNQGVPARKFASTVVDPFGKGKTQHDSLDDESISQSSSIKQRELNAADKC |
| S. cerevisiae ACY185 | MKNIISLVSKKKAASKNEDKNISESSRDIVNQQEVFNTENFEEGKKDSAFELDHLEFTTNSAQLGDSDEDNENVINETNTTDDANEANSEEKSMTLKQALLIYPKAALWSILVSTTLVMEGYDTALLNALYALPVFQRKFGTLNGEGSYEITSQWQIGLNMCVQCGEMIGLQITPYMVEFMGNRYTMITALGLLTAYVFILYYCKSLAMIAVGQVLSAMPWGCFQGLTVTYASEVCPLALRYYMTSYSNICWLFGQIFASGIMKNSQENLGNSDLGYKLPFALQWIWPAPLMIGIFFAPESPWWLVRKDRVAEARKSLSRILSGKGAEKDIQIDLTLKQIELTIEKERLLASKSGSFFDCFKGVNGRRTRLACLTWVAQNTSGACLLGYSTYFFERAGMATDKAFTFSVIQYCLGLAGTLCSWVISGRVGRWTILTYGLAFQMVCLFIIGGMGFGSGSGASNGAGGLLLALSFFYNAGIGAVVYCIVTEIPSAELRTKTIVLARICYNIMAVINAILTPYMLNVSDWNWGAKTGLYWGGFTAVTLAWVIIDLPETSGRTFSEINELFNQGVPARKFASTVVDPFGKGKTQHDSLADESISQSSSIKQRELNAADKC |
| S. cerevisiae ACY8/ACY9/ACY20/ACY29 | MKNIISLVSKKKAASKNEDKNISESSRDIVNQQEVFNTEDFEEGKKDSAFELDHLEFTTNSAQLGDSDEDNENVINEMNATDDANEANSEEKSMTLKQALLKYPKAALWSILVSTTLVMEGYDTALLSALYALPVFQRKFGTLNGEGSYEITSQWQIGLNMCVLCGEMIGLQITTYMVEFMGNRYTMITALGLLTAYIFILYYCKSLAMIAVGQILSAIPWGCFQSLAVTYASEVCPLALRYYMTSYSNICWLFGQIFASGIMKNSQENLGNSDLGYKLPFALQWIWPAPLMIGIFFAPESPWWLVRKDRVAEARKSLSRILSGKGAEKDIQVDLTLKQIELTIEKERLLASKSGSFFNCFKGVNGRRTRLACLTWVAQNSSGAVLLGYSTYFFERAGMATDKAFTFSLIQYCLGLAGTLCSWVISGRVGRWTILTYGLAFQMVCLFIIGGMGFGSGSSASNGAGGLLLALSFFYNAGIGAVVYCIVAEIPSAELRTKTIVLARICYNLMAVINAILTPYMLNVSDWNWGAKTGLYWGGFTAVTLAWVIIDLPETTGRTFSEINELFNQGVPARKFASTVVDPFGKGKTQHDSLADESISQSSSIKQRELNAADKC |

## Slide 11
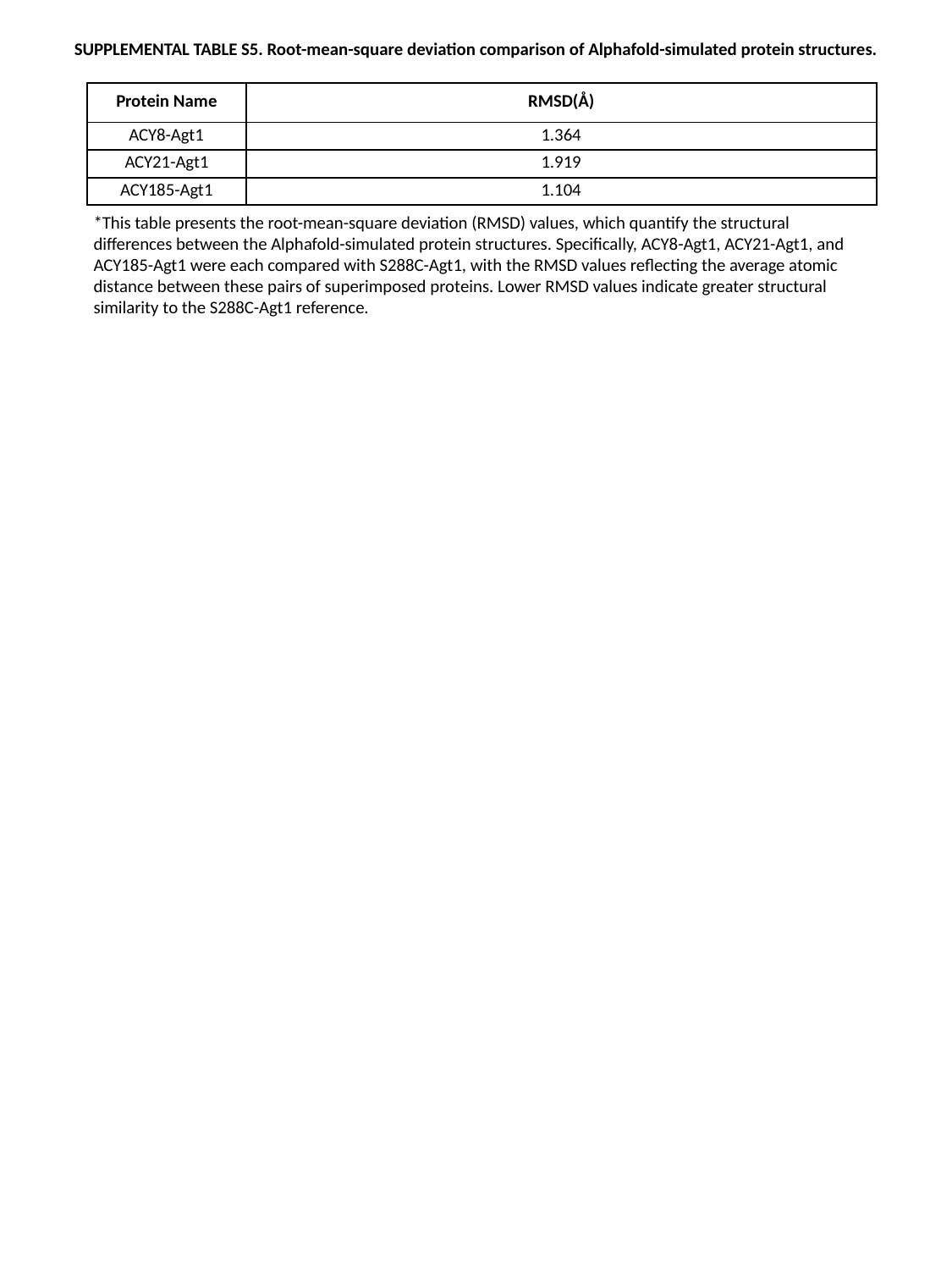

SUPPLEMENTAL TABLE S5. Root-mean-square deviation comparison of Alphafold-simulated protein structures.
| Protein Name | RMSD(Å) |
| --- | --- |
| ACY8-Agt1 | 1.364 |
| ACY21-Agt1 | 1.919 |
| ACY185-Agt1 | 1.104 |
*This table presents the root-mean-square deviation (RMSD) values, which quantify the structural differences between the Alphafold-simulated protein structures. Specifically, ACY8-Agt1, ACY21-Agt1, and ACY185-Agt1 were each compared with S288C-Agt1, with the RMSD values reflecting the average atomic distance between these pairs of superimposed proteins. Lower RMSD values indicate greater structural similarity to the S288C-Agt1 reference.

## Slide 12
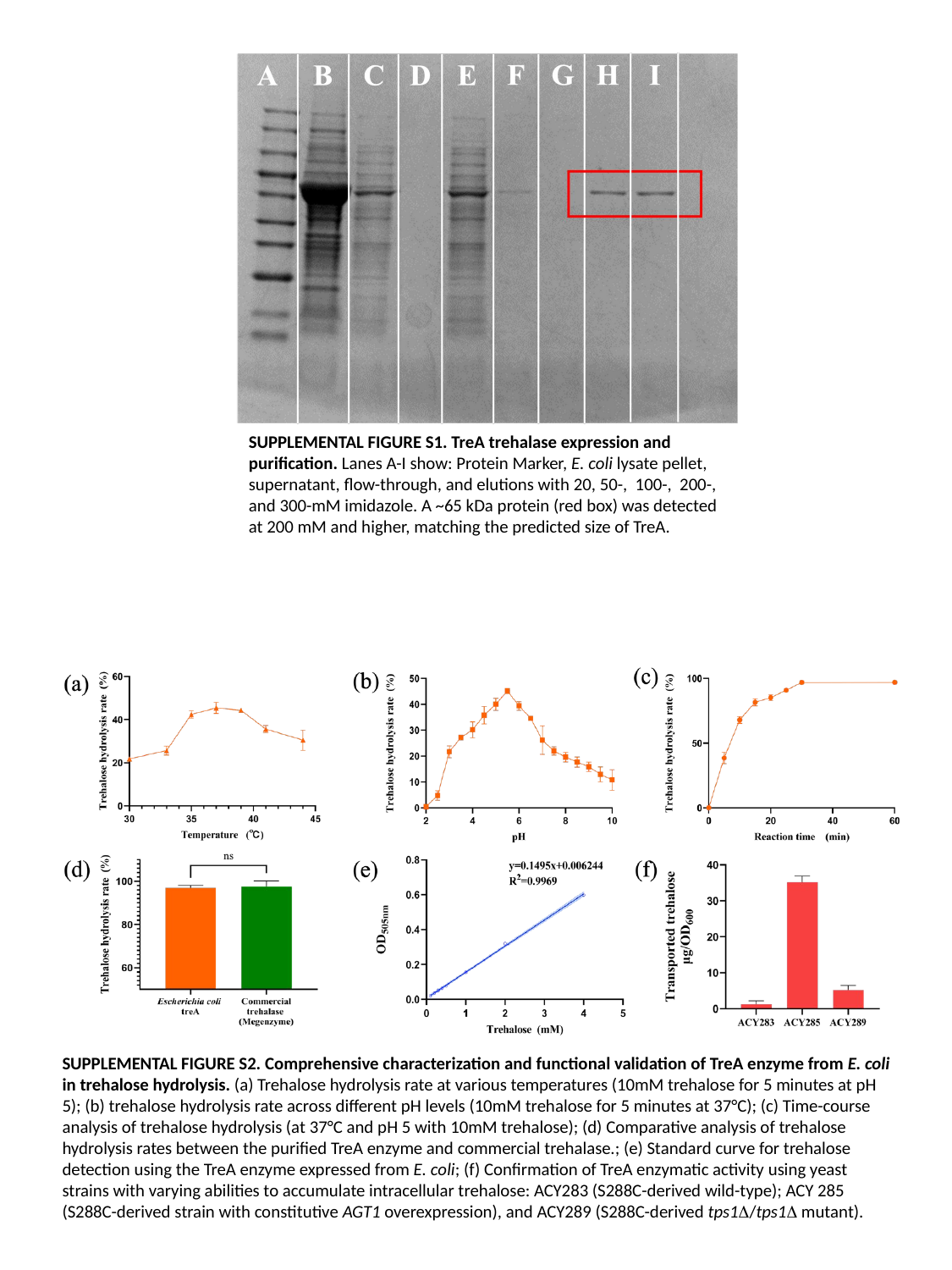

SUPPLEMENTAL FIGURE S1. TreA trehalase expression and purification. Lanes A-I show: Protein Marker, E. coli lysate pellet, supernatant, flow-through, and elutions with 20, 50-, 100-, 200-, and 300-mM imidazole. A ~65 kDa protein (red box) was detected at 200 mM and higher, matching the predicted size of TreA.
SUPPLEMENTAL FIGURE S2. Comprehensive characterization and functional validation of TreA enzyme from E. coli in trehalose hydrolysis. (a) Trehalose hydrolysis rate at various temperatures (10mM trehalose for 5 minutes at pH 5); (b) trehalose hydrolysis rate across different pH levels (10mM trehalose for 5 minutes at 37°C); (c) Time-course analysis of trehalose hydrolysis (at 37°C and pH 5 with 10mM trehalose); (d) Comparative analysis of trehalose hydrolysis rates between the purified TreA enzyme and commercial trehalase.; (e) Standard curve for trehalose detection using the TreA enzyme expressed from E. coli; (f) Confirmation of TreA enzymatic activity using yeast strains with varying abilities to accumulate intracellular trehalose: ACY283 (S288C-derived wild-type); ACY 285 (S288C-derived strain with constitutive AGT1 overexpression), and ACY289 (S288C-derived tps1Δ/tps1Δ mutant).

## Slide 13
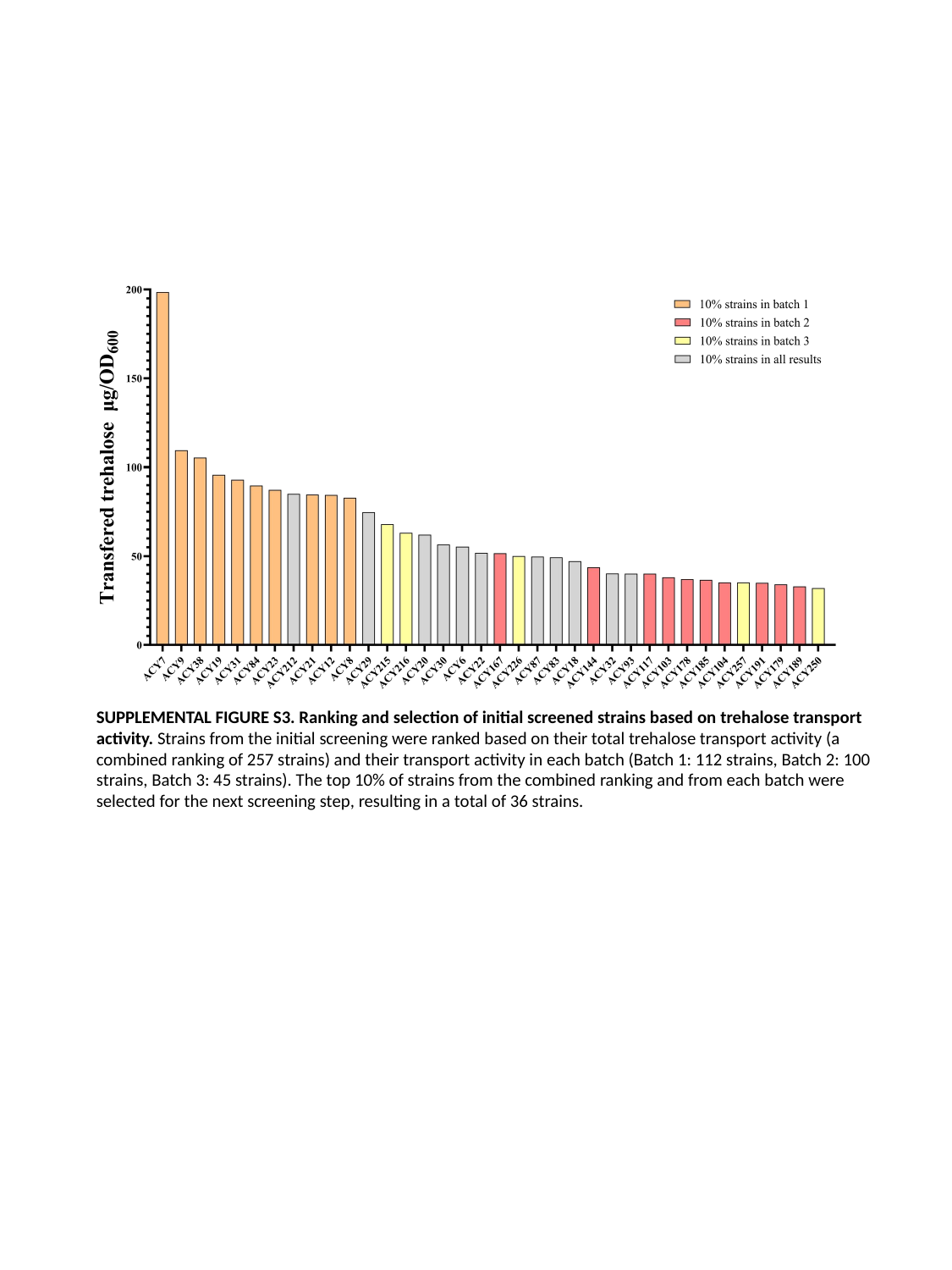

SUPPLEMENTAL FIGURE S3. Ranking and selection of initial screened strains based on trehalose transport activity. Strains from the initial screening were ranked based on their total trehalose transport activity (a combined ranking of 257 strains) and their transport activity in each batch (Batch 1: 112 strains, Batch 2: 100 strains, Batch 3: 45 strains). The top 10% of strains from the combined ranking and from each batch were selected for the next screening step, resulting in a total of 36 strains.

## Slide 14
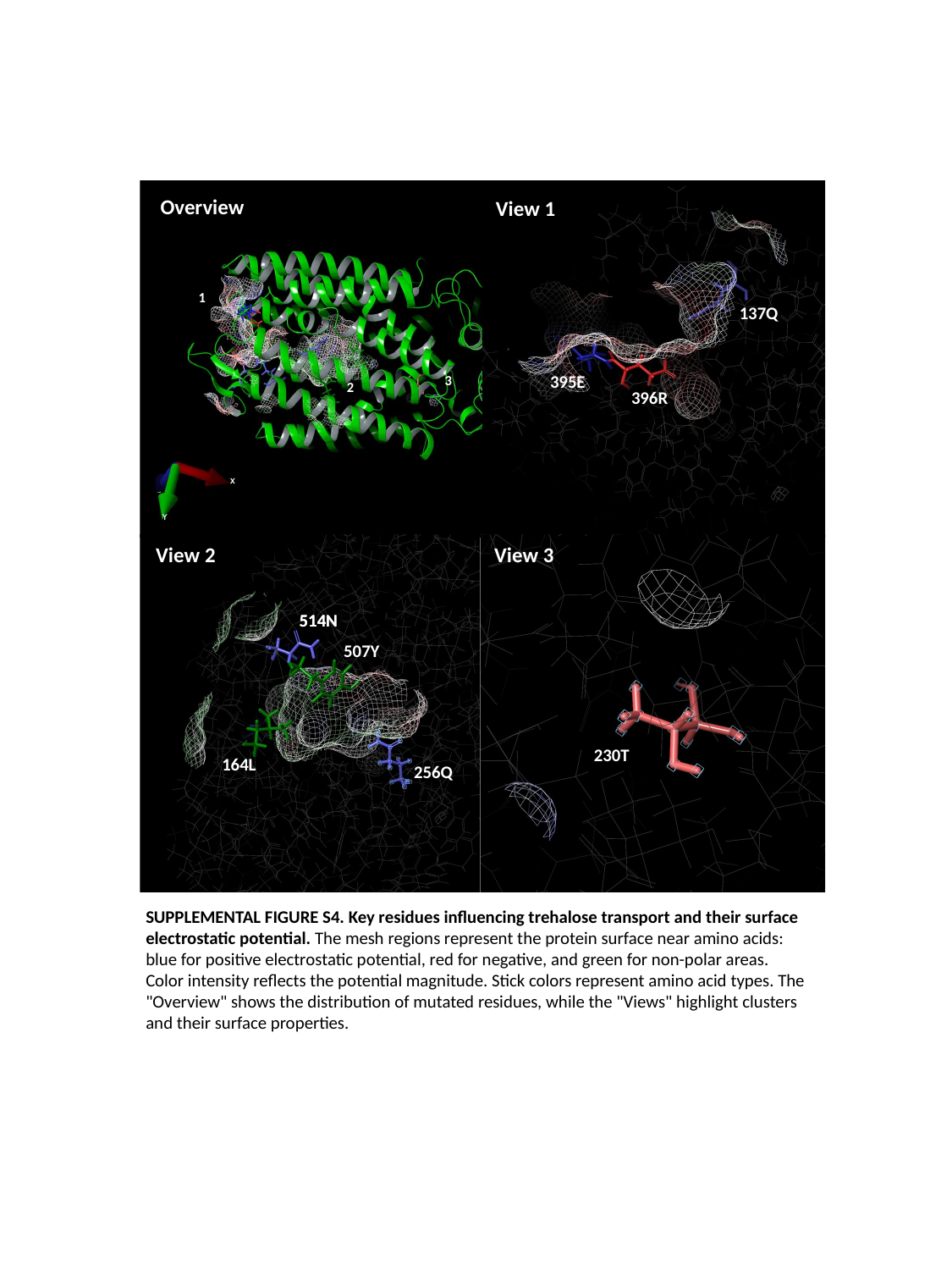

View 1
Overview
1
137Q
395E
3
2
396R
View 2
View 3
514N
514N
507Y
230T
164L
256Q
SUPPLEMENTAL FIGURE S4. Key residues influencing trehalose transport and their surface electrostatic potential. The mesh regions represent the protein surface near amino acids: blue for positive electrostatic potential, red for negative, and green for non-polar areas. Color intensity reflects the potential magnitude. Stick colors represent amino acid types. The "Overview" shows the distribution of mutated residues, while the "Views" highlight clusters and their surface properties.
